# The role of surgery on the primary tumor site in bladder cancer with distant metastasis: significance of histology type and metastatic pattern

**DOI:** 10.1002/cam4.3560

**Published:** 2020-10-27

**Authors:** Ping Wang, Shuang Zang, Guangqi Li, Weiling Qu, Shuyao Li, Qiao Qiao, Yuanjun Jiang

**Affiliations:** ^1^ Department of Radiation Oncology the First Hospital of China Medical University Shenyang China; ^2^ School of Nursing China Medical University Shenyang Liaoning China; ^3^ Department of Urology the First Hospital of China Medical University Shenyang Liaoning China

**Keywords:** bladder cancer, distant metastasis, PSM, SEER, surgery

## Abstract

Due to the limited data and research on bladder cancer with distant metastasis, the role of surgery on the primary tumor site in metastatic bladder cancer has been controversial. The aim of this study was to investigate the impact of surgery on patients with metastatic bladder cancer and to identify any factors correlated with the treatment efficacy. Using the Surveillance, Epidemiology and End Results dataset, we performed a large population‐based retrospective study. We classified patents with distant metastasis into subgroups according to their histology type and metastatic pattern. Propensity score matching (PSM) was used to balance clinical variables bias in a 1:3 ratio. A total of 2470 patients with distant metastasis were identified from 2010 to 2016. After PSM, the study eventually included 1068 patients in the surgery group and 356 patients in the non‐surgery group. The histologic types, the number of metastatic sites, liver metastasis, surgery, and chemotherapy were significant prognostic variables for patients with distant metastasis before and after PSM. In terms of histologic types, the survival of patients with transitional cell papillary carcinoma, transitional cell non‐papillary carcinoma and adenocarcinoma can be improved by surgery alone, while the survival of patients with squamous cell carcinoma can be improved only by combining chemotherapy and surgery. In terms of the number of metastatic sites, surgery alone was an independent prognostic factor in patients with 1 or 2 metastatic sites. In terms of the specificity of metastatic organs, surgery affected overall survival for patients with bone metastasis only, liver metastasis only and lung metastasis only, but for distant lymph nodes metastasis only. It may be helpful to classify patients with bladder cancer and distant metastasis into different groups by integrating variables including histology types and metastatic patterns to choose appropriate treatment strategies.

## Graphical abstract

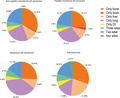

## 1 INTRODUCTION

Bladder cancer is among the most common urinary malignancies.^1^ Every year, 430000 new cases are diagnosed and 165000 bladder cancer related‐deaths occur globally.^2^ Up to 50% of patients with muscle‐invasive bladder cancer develop distant metastasis to the lymph nodes, lungs, liver, and bone.^3^ Additionally, distant metastasis has already occurred at the time of diagnosis in 10%‐15% of bladder cancers.^4^ Untreated patients with distant metastasis generally have poor prognoses, and their median overall survival rarely exceeds 3 to 6 months. With standard treatment of chemotherapy, the median survival time is still only 15 months.^5^, ^6^, ^7^ According to the current guidelines, more than 50% of patients are not suitable for chemotherapy, although chemotherapy is still used as first‐line treatment. The role of surgery as a second‐line treatment remains unclear.^3^ There are few studies suggesting that patients with distant metastases benefit from surgery, especially primary tumor resection, when metastasis is present at the time of diagnosis of bladder cancer.^8^, ^9^

The most common histological type of bladder cancer is transitional cell carcinoma, accounting for almost 90% of all urothelial carcinomas. It can be further classified into transitional cell papillary carcinoma (TCPC) and transitional cell non‐papillary carcinoma (TCC). The remaining 10% of bladder cancers are 1.2 to 5% squamous cell carcinoma (SCC), 0.5 to 2% adenocarcinoma and <1% sarcoma.^10^ Patients with TCPC and TCC of the bladder have a better prognosis than those with SCC, adenocarcinomas and sarcomas.^11^ The histological type of bladder cancer affects not only the prognosis but also the therapeutic efficacy.^12^

The most common sites of distant metastasis include lymph nodes (25.4%), bone (24.7%), urinary tract (23.5%), lung (19.4%), liver (18.1%), and brain (3.1%).^13^ Both the histology and metastatic pattern are associated with the prognosis in metastatic bladder cancer.^14^

Population‐based analyses on the role of surgery according to histology types and metastatic patterns are deficient. Thus, the main purpose of our study was to investigate the role of surgery using patient stratification by histology type and metastatic pattern.

## 2 MATERIALS AND METHODS

### 2.1 Patient selection

We conducted a retrospective, population‐based study with data from the Surveillance, Epidemiology, and End Results (SEER) national database. The following inclusion and exclusion criteria were applied.

Inclusion criteria: (1) Patients were diagnosed with bladder cancer between 2010 and 2016. (2) Bladder cancer was the first primary malignancy.

Exclusion criteria: information about age, sex, race, primary site, histologic type, tumor grade, surgery, radiotherapy, chemotherapy, metastatic information, survival time, and current status were unavailable.

### 2.2 Study variables and endpoints

The variables including the age at diagnosis, sex, race, primary site, histologic type, grade, treatment, seventh edition of the AJCC Staging Manual tumor stage including Ta/Tis/T1/T2a/T2b/T3a/T4b/N0/N1/N2/N3/M0/M1 stage, sites of distant metastases and the number of distant metastatic sites were analysed in our research. We used the pathological grading standard according to the SEER database and previous studies.^15^, ^16^ The data for metastasis to distant lymph nodes, liver, lung, bone, and brain were identified at the time of diagnosis. The overall survival (OS) was considered as the endpoint.

### 2.3 Statistical analyses

Survival times were compared by Kaplan‐Meier methods and log‐rank tests. Independent prognostic variables associated with OS were identified by univariate and multivariate Cox regression analyses. We set a 1:3 ratio to reduce bias by the “MatchIt” R package using a propensity score matching (PSM) method. Student's t‐test was used to make comparisons for continuous variables. The Chi‐square test and Fisher's exact test were used to make comparisons for categorical variables. Odds ratios were calculated to describe the correlation relationships between different metastatic sites. A *p* value <0.05 was considered statistically significant in all analyses. We used R version 3.6.1, IBM SPSS Statistics software version 26 and GraphPad Prism version 8 to perform all statistical analyses.

## 3 RESULTS

### 3.1 Patient characteristics and poor prognosis predicted by distant metastasis.

We enrolled 91744 patients diagnosed with bladder cancer. Among the included patients, 88184 (96.12%) patients had transitional cell carcinoma, including 63863 (69.61%) papillary transitional cell carcinomas and 24321 (26.51%) non‐papillary transitional cell carcinomas, while 1437 (1.57%) patients had squamous cell carcinoma, 716 (0.78%) patients had adenocarcinoma, and 1407 (1.53%) had other types not otherwise specified (NOS).

Among the final cohort, 2470 (2.69%) were recorded as having distant metastasis at the time of diagnosis. The parameters with differences between the metastatic group and the non‐metastatic group are shown in Table S1. Compared to the non‐metastatic group, the metastatic group had a higher incidence of patients 40–60 years old, female sex, black and other race, a higher grade and a higher rate of non‐papillary transitional cell carcinoma, squamous cell neoplasms and adenocarcinomas. In terms of treatment, the patients with distant metastasis were less likely to undergo surgery but more likely to receive chemotherapy and radiotherapy compared to those without distant metastasis.

**Figure 1:**
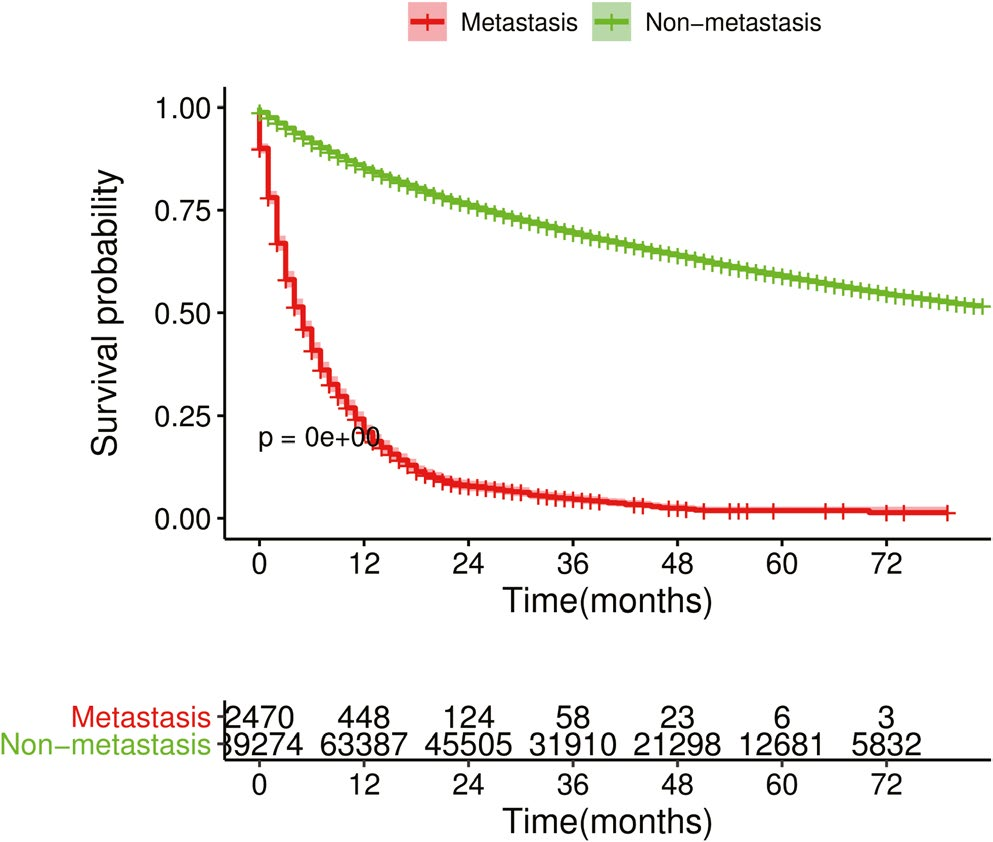
Kaplan‐Meier survival curves for bladder cancer patients with and without distant metastasis

Furthermore, we found the metastasis group had a significantly worse survival than the non‐metastasis group through Kaplan‐Meier curves (*p* < 0.0001, Figure 1). Univariable and multivariable Cox regression model analyses also demonstrated that distant metastasis was an independent prognostic factor for OS (Table S2).

### 3.2 Metastatic patterns and histology types

In all 2470 patients with distant metastasis, the most common sites were bone (1183, 47.89%), followed by lung (1140, 46.15%), liver (718, 29.07%), distant lymph nodes (198, 8.01%), and brain (85, 3.44%). Most patients (1784, 72.23%) had a single site of distant metastasis, followed by two sites (533, 21.58%), three sites (138, 5.59%) and four sites (15, 0.61%). Details of the distributions of the distant metastatic sites are presented in Table 1.

**Table 1:** Detailed distributions of distant metastatic sites

Sites of distant metastases	*N* (%)
One site of distant metastasis	
Bone	711(28.79%)
Lung	632(25.59%)
Liver	306(12.39%)
Brain	30(1.21%)
Distant lymph node	105(4.25%)
Two sites of distant metastasis	
Bone +lung	182(7.37%)
Liver +lung	141(5.71%)
Bone +liver	120(4.86%)
Brain +lung	15(0.61%)
Bone +brain	12(0.49%)
Brain +liver	2(0.08%)
Distant lymph node+bone	23(0.93%)
Distant lymph node+liver	10(0.40%)
Distant lymph node +lung	28(1.13%)
Three sites of distant metastasis	
Liver+lung +bone	96(3.89%)
Brain +liver + lung	8(0.32%)
Bone +brain + lung	6(0.24%)
Bone +brain + liver	4(0.16%)
Distant lymph node+Liver+ lung	10(0.40%)
Distant lymph node+lung +bone	8(0.32%)
Distant lymph node+liver +bone	6(0.24%)
Four sites of distant metastasis	
Liver +lung + brain +bone	7(0.28%)
Liver +lung + bone +Distant lymph node	7(0.28%)
Distant lymph node +brain + bone +liver	1(0.04%)

In addition, we further compared the differences in metastasis patterns among the different histology types. For the TCC and TCPC, bone was the leading lesion as a single metastatic site, followed by lung, liver, and brain. There were no brain metastases from the squamous cell carcinomas and adenocarcinomas. Lung was the leading lesion as a single metastatic site in squamous cell carcinoma, followed by bone, and liver, whereas liver was the leading lesion as a single metastatic site in adenocarcinoma, followed by bone and lung (Figure S1).

As for multiple‐sites metastases, bi‐site pattern(TCC:20.96%,TCPC:22.12%,SCC:15.73%,adenocarcinoma:18.07%) was significantly higher than three‐sites patterns(TCC:5.79%,TCPC:4.48%,SCC:6.74%, adenocarcinoma:7.23%) and four‐sites patterns(TCC:0.56%,TCPC:0.45%,SCC:0%, adenocarcinoma:2.61%). Furthermore, lung metastasis was more likely to co‐metastasize with a brain metastasis (OR: 60.329) or a liver metastasis (OR: 61.932) than with other metastases (Figure S2).

### 3.3 Prognostic Factors For 2470 metastatic cancers before and after PSM

To evaluate the effect of surgery on the outcome more accurately, we apply PSM to unify the background of the patients with and without surgery. The clinicopathological features of the two groups before and after PSM are shown in Table 2. After PSM, the differences of N stage, chemotherapy and the number of metastatic sites were balanced, and the differences of histology types, grade, and T stage were decreased although still significant.

**Table 2:** Clinicopathological features with and without surgery before and after propensity score matching

Variables	No PSM			PSM		
	Surgery (*n* = 2114)	No‐surgery (*n* = 356)	*p* value	Surgery（n = 1068）	No‐surgery (n = 356)	*p* value
Age at diagnosis(years)	70.91(17‐100)	70.79(28‐98)	0.680	71.40(17‐100)	70.79(28‐98)	0.412
Race			0.067			0.491
White	1783	287		827	287	
Black	226	53		137	53	
Other	105	16		59	16	
Sex			0.519			0.920
Female	589	105		320	105	
Male	1529	251		748	251	
Bladder, NOS						
Histologic type			**<0.0001**			**<0.0001**
Non‐Papillary Transitional cell carcinoma	1202	215		557	215	
Papillary Transitional cell carcinoma	615	54		313	54	
Squamous cell neoplasms	68	21		52	21	
Adenomas and adenocarcinoma	69	14		47	14	
Other	160	52		99	52	
Grade			**<0.0001**			**<0.0001**
II	107	24		95	24	
III	519	147		328	147	
IV	1488	185		645	185	
T stage			**<0.0001**			**<0.0001**
T0	0	7		0	7	
T1	289	55		119	55	
T2	841	50		299	50	
T3	142	20		68	20	
T4	303	65		208	65	
TX +unknown	539	159		374	159	
N stage			**0.011**			0.665
N0	1008	134		435	134	
N1	152	28		82	28	
N2	275	53		131	53	
N3	80	15		38	15	
NX +unknown	599	126		382	126	
Radiotherapy			0.435			0.614
Refused	23	2		12	2	
Yes	496	75		215	75	
None/Unknown	1595	279		341	279	
Chemotherapy			**<0.0001**			0.896
No/Unknown	1039	243		723	243	
Yes	1075	113		345	113	
Number of sites of metastases	1.33(1‐4)	1.42(1‐4)	**0.036**	1.40	1.42	0.695

The *p* value was bold when it <0.05.

**Figure 2:**
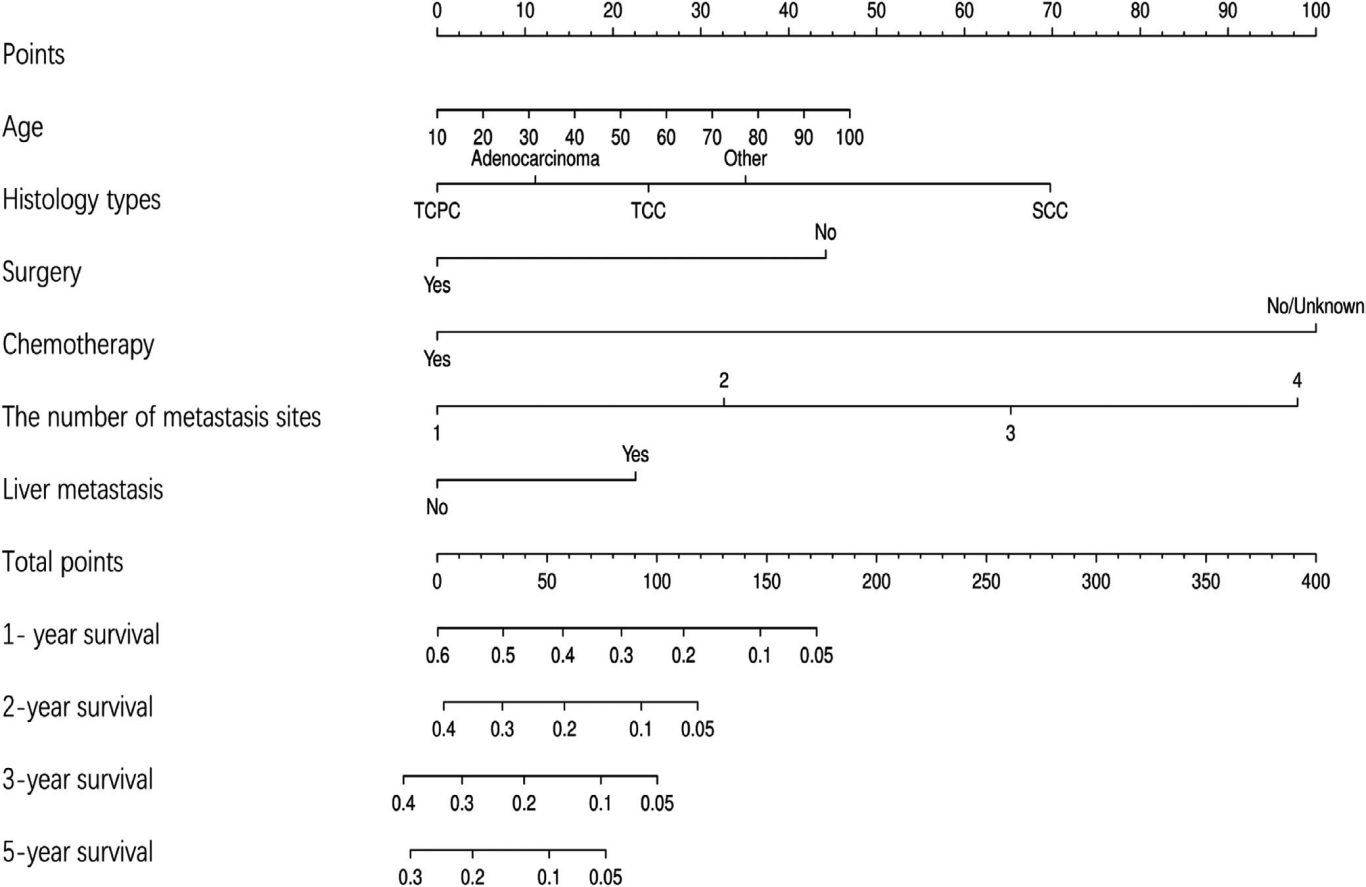
Nomogram predicting 1, 2, 3 and 5 years overall survival in bladder cancer with distant metastasis

Univariate and multivariable Cox analysis revealed that age, histology type, and treatment methods including surgery, LNs removed, chemotherapy, the metastatic site and the number of metastatic sites were associated with overall survival before PSM. Moreover, these factors were still significant in univariate and multivariable Cox analysis after PSM (Table 3). The patients with squamous cell carcinoma, non‐papillary transitional cell carcinoma and other types had a worse survival than those with papillary transitional cell carcinoma and adenocarcinoma(Figure S3A). For metastatic sites, multiple metastases resulted in a worse prognosis than single site metastasis, and besides, liver metastasis had the worst prognosis among the metastatic bladder cancers (Figure S3B,C). We also constructed a nomogram to predict survival within 5 years according to these 5 independent prognostic factors (Figure 2). The AUC for predicting 1, 2.3, and 5 years OS were 0.776, 0.726, 0.711, and 0.686, respectively.

**Table 3:** Univariable and multivariable Cox regression model analyses of overall survival in 1424 distant metastatic bladder cancers after PSM

Variables	level	Univariable			multivariable		
		*p*	HR	95% CI	*p*	HR	95% CI
Age at diagnosis(years)	<70	Ref					
	>=70	**<0.0001**	1.284	1.144‐1.439	**0.044**	1.130	1.003‐1.272
Race	White	Ref					
	Black	0.351	1.081	0.918‐1.272			
	Other	0.579	0.927	0.710‐1.211			
Sex	Female	Ref					
	Male	**0.004**	0.836	0.740‐0.946	0.442	0.952	0.840‐1.078
Primary Site	Trigone of bladder	Ref					
	Dome of bladder	0.096	0.661	0.406‐1.077			
	Lateral wall of bladder	0.852	1.032	0.738‐1.444			
	Anterior wall of bladder	0.253	0.756	0.468‐1.221			
	Posterior wall of bladder	0.389	1.171	0.817‐1.678			
	Bladder neck	0.289	0.806	0.541‐1.201			
	Ureteric orifice	0.206	0.714	0.424‐1.204			
	Ureteric orifice	**0.014**	0.317	0.127‐0.792			
	Overlapping lesion of bladder	0.759	0.955	0.711‐1.282			
	Bladder, NOS	0.779	0.961	0.730‐1.266			
Histologic type	Non‐Papillary Transitional cell carcinoma	Ref					
	Papillary Transitional cell carcinoma	**<0.0001**	0.768	0.669‐0.882	**0.001**	0.797	0.693‐0.916
	Squamous cell neoplasms	**0.005**	1.436	1.118‐1.844	**0.001**	1.575	1.220‐2.034
	Adenomas and adenocarcinoma	**0.010**	0.684	0.512‐0.913	0.308	0.860	0.969‐1.412
	Other	0.117	1.158	0.964‐1.391	0.102	01.170	0.601‐0.781
Grade	II	Ref					
	III	0.142	1.176	0.947‐1.461			
	IV	0.654	1.049	0.851‐1.293			
T stage	T0	Ref					
	T1	0.654	1.205	0.533‐2.726			
	T2	0.513	1.310	0.583‐2.942			
	T3	0.727	1.159	0.505‐2.660			
	T4	0.575	1.262	0.560‐2.840			
	TX +unknown	0.490	1.330	0.592‐2.992			
N stage	N0	Ref					
	N1	0.951	1.007	0.816‐1.242			
	N2	0.962	1.004	0.847‐1.191			
	N3	0.329	1.152	0.867‐1.532			
	NX +unknown	0.962	1.012	0.881‐1.163			
Surgery	No	Ref					
	Yes	**<0.0001**	0.710	0.625‐0.806	**<0.0001**	0.685	0.601‐0.849
Surgery about regional lymph nodes	Surgery but no LNs removed	Ref					
	No surgery	**<0.0001**	1.348	1.186‐1.532			
	Surgery and LN removed	**<0.0001**	0.601	0.464‐0.779	**<0.0001**	0.374	0.327‐0.428
Radiotherapy	Refused	Ref					
	Yes	0.356	.0.761	0.426‐1.359			
	None/Unknown	0.541	0.837	0.473‐1.480			
Radiation sequence with surgery	No radiotherapy and/or surgery	Ref					
	Radiation after surgery	0.082	0.865	0.735‐1.019			
	Radiation before surgery	0.115	0.524	0.395‐1.747			
	other	0.624	0.830	0.395‐1.747			
Chemotherapy	No/Unknown	Ref					
	Yes	**<0.0001**	0.408	0.359‐0.463	**<0.0001**	0.374	0.327‐0.428
Number of sites of metastases	1	Ref					
	2	0.064	1.137	0.992‐1.302	**0.001**	1.267	1.096‐1.465
	3	**<0.0001**	1.805	1.444‐2.257	**<0.0001**	1.753	1.359‐2.262
	4	0.148	1.523	0.861‐2.694	0.129	1.630	0.868‐3.063
Metastases sites including liver	No	Ref					
	Yes	**<0.0001**	1.407	1.247‐1.586	**0.001**	1.260	1.098‐1.446
Metastases sites including brain	No	Ref					
	Yes	**0.041**	1.361	1.013‐1.827	0.259	1.210	0.869‐1.688

The *p* value was bold when it <0.05.

### 3.4 OS analysis classified by histology types after PSM

For patients with non‐papillary transitional cell carcinoma, univariate and multivariable Cox regression analysis revealed grade, surgery, chemotherapy and the number of metastatic sites and metastatic sites including liver were independent prognostic factors of overall survival (Table S3). However, the LNs removed was not an independent prognostic factor among patients with surgery and there were no significant differences in survival for patients with surgery with and without LNs removed (Figure S4A).

For patients with papillary transitional cell carcinoma, univariate and multivariable Cox regression analysis found that age, chemotherapy, LNs removed, the number of metastatic sites and metastatic sites including liver were prognostic factors for overall survival (Table S4). Of note, only surgery without LNs removed was not associated with improved patient survival (Figure S4B).

However, neither surgery nor LNs removed affected the overall survival of patients with squamous cell carcinoma and adenocarcinoma. Only chemotherapy and the number of metastatic sites and metastatic sites including liver were significant prognostic indicators for OS of patients with squamous cell carcinoma and adenocarcinoma, respectively (Tables S5, S6).

### 3.5 OS analysis classified by the number of metastatic sites after PSM

Univariate and multivariate Cox analysis for patients with one site of distant metastasis indicated that age, histologic types, surgery, LNs removed and chemotherapy were independent prognostic factors for OS (Table S7). Patients with LNs removed had a better prognosis than those without LNs removed or surgery (*p* < 0.0001, Figure S5).

For patients with two sites of distant metastasis, surgery, chemotherapy and metastatic sites other than liver were independent predictive factors for a longer OS (Table S8). There were no significant differences in the survival of patients with and without LNs removed (*p* = 0.905).

Nevertheless, only chemotherapy was an independent prognostic factor for OS in patients with three to four sites of distant metastasis (Table S9).

### 3.6 OS analysis classified by specific organ for patients with one site metastasis after PSM

For patients with bone metastasis only, histology type, surgery, LNs removed and chemotherapy were associated with overall survival in univariate and multivariate Cox analysis (Table S10). Patients with LNs removed had a better prognosis than those without LNs removed or surgery (*p* < 0.0001, Figure S6A).

For patients with lung metastasis, univariable and multivariable Cox regression model analyses found that histology type, surgery, LNs removed and chemotherapy were independent prognostic factors (Table S11). Patients with LNs removed had a better prognosis than those without LNs removed or surgery (*p* < 0.0001, Figure S6B).

For patients with liver metastasis, age, surgery, and chemotherapy were independent prognostic factors. However, LNs removed in surgery was not an independent prognostic factor (Table S12).

For patients with DL metastasis, neither surgery nor lymph node removal affected their prognosis, but histology types and chemotherapy were independent prognostic factors (Table S13).

### 3.7 Comparison of treatment effect according to histology types and metastatic patterns

To investigate if there was an interaction between the two kinds of treatment including surgery and chemotherapy, we further analysed the effects of surgery and chemotherapy in different patient stratifications. For histological stratification, surgery alone can improve the prognosis of patients with TCPC and TCC, while chemotherapy alone can improve the prognosis of patients with TCPC, TCC and adenocarcinoma, and patients with TCC who had chemotherapy alone had a better survival than those with surgery alone (Figure S7A‐C). However, only a combination of surgery and chemotherapy could improve patient survival of SCC (Figure S7D).

According to stratification by the number of metastatic sites, although both chemotherapy and surgery could improve the prognosis, the effect of chemotherapy was significantly better than surgery in patients with 1–2 sites of distant metastasis (Figure S7E‐F). For patients with 3–4 sites of distant metastasis, the effect of a combination of chemotherapy and surgery was significantly better than chemotherapy alone (Figure S7G). In the terms of specific sites in patients with one site of distant metastasis, surgery alone and chemotherapy alone improved the prognosis of patients with bone, lung and liver metastasis, but patients with bone and lung metastasis treated by chemotherapy alone had a better prognosis than those with surgery alone (Figure S7H,I,J). Nevertheless, patients with DL metastasis only benefited from a combination of chemotherapy and surgery (Figure S7K).

## 4 DISCUSSION

Surgical removal of the primary tumor is an integral part of multimodal treatment in many metastatic urological and nonurological cancers.^17^, ^18^ However, only a few studies have investigated the effect of surgery on survival outcomes in metastatic bladder cancer.^19^, ^20^ This large sample, population‐based study, is to our knowledge the first study to explore the prognostic factors for overall survival of patients with bladder cancer and distant metastasis based on their histologic types and metastatic patterns, and this study has provided important information for clinical decision making. Our research has indicated that histology type, the specific metastatic site and the number of metastatic sites are independent prognostic factors for bladder cancer with distant metastasis. Moreover the treatment including surgery, LNs removed, radiotherapy and chemotherapy for bladder cancer with distant metastasis were studied according to the histology types, the specific metastatic sites and the number of metastatic sites.

Our research showed that the patients with distant metastasis at the time of diagnosis had a worse prognosis compared to those without distant metastasis. To assess the risk of distant metastasis, we further compared the clinical parameters between the metastatic group and non‐metastatic group. We found the histologic types TCC, SCC, and adenocarcinoma were risk factors for distant metastasis compared with other types. Similarly, Atul B. Shinagare et al. also demonstrated the TCC is the most common histologic type in metastatic bladder cancer.^21^

Although bladder cancers are more common in men than in women, women with bladder cancer are often at a late stage at the time of diagnosis.^2^ The rate of cancer metastasis and recurrence also increases as the tumor grade increases, as shown in previous studies.^22^ Consistent with these results, patients who were 40–60 years old, female sex, black race, and high grade were more likely to have a distant metastasis in our study. Therefore, it is necessary to examine and follow‐up these populations regularly.

In our research, bone was the most common site of distant metastasis, followed by lung, liver, brain, and LN. Similarly, Bianchi et al.^13^ found a higher bone metastasis rate than to lung and liver for bladder cancer with distant metastasis. Histology types are associated with the metastasis pattern in lung cancer;^23^ however, studies about the relationship between histology types and metastatic pattern are deficient for bladder cancer. Our research suggested that the metastasis pattern depended on histology type. Bone was the leading lesion in transitional cell carcinoma, whereas lung and liver were the leading lesions in squamous cell neoplasms and adenocarcinoma. Multiple metastases are more likely to occur in adenocarcinoma compared to other histology types.

According to results from previous studies,^4^, ^24^ chemotherapy has always been regarded as the gold standard for the treatment of metastatic cancer of the bladder, but there are only a few previous reports on the role of surgery. Of note, our research demonstrated that surgery and chemotherapy were independent prognostic factors for overall survival in bladder cancers with distant metastasis in univariate and multivariable Cox regression analysis. In addition, the histologic types, liver metastasis and the number of metastatic sites were also independent prognostic factors for overall survival in bladder cancers with distant metastasis. The efficacy of surgery for the primary tumor is related to the number of metastatic sites,^25^ and we made an important addition to this conclusion by stratifying patients into different subgroups according to their histologic types, metastasis sites and the number of metastatic sites.

In terms of histologic types, patients with TCC, TCPC, and adenocarcinoma do well with chemotherapy alone; however, patients with SCC only benefit from a combination of surgery with chemotherapy. In terms of metastasis sites, chemotherapy alone is suitable for patients with ≤2 sites of metastasis, and a combination of surgery with chemotherapy benefits only patients with >2 sites of metastasis. Even in cases of one‐site metastasis, patients with lymph node metastasis should be treated by combining chemotherapy and surgery. There is previous evidence that lymph nodes dissection has survival benefits for upper tract urothelial carcinoma,^26^ and our results suggested that surgery with LNs removed in surgery improved the overall survival of patients with TCPC and one site of metastasis.

Though we performed this research rigorously, there are still many limitations. First, the inclusion times for the DL metastases are not consistent with the other four sites in the SEER dataset. Second, the SEER database lacks other important information about smoking status, and the order of surgery and chemotherapy is unknown. Third, our experiment did not eliminate all variable bias between the surgery and non‐surgery group after PSM.

## 5 CONCLUSION

We performed a large‐scale retrospective study about the role of surgery on the primary site in the treatment of metastatic bladder cancer. Distant metastasis is an independent prognostic factor for overall survival in bladder cancer. The metastatic patterns are significantly different among different histological types. Surgery has different impacts on the survival outcomes of patients with distant metastasis based on their different histology types and metastatic patterns. Although surgery alone can improve patient prognosis, its efficacy is worse than chemotherapy alone for most patients, including patients with TCC, adenocarcinomas, ≤2 sites of metastasis, and lung only and bone only metastasis. However, for some patients, including those with SCC, >2 sites of metastasis, or DL metastasis only, it is important to combine surgery and chemotherapy to improve the patient's prognosis. Thus, it is helpful to evaluate the role of surgery according to patient stratification based on individual histologic type and metastatic pattern.

## CONFLICTS OF INTEREST

The authors declare no potential conflicts of interest.

## ETHICAL APPROVAL

All data were from the SEER database. The SEER database is publicly available and considered.

## Supporting information

Fig S1

Fig S2

Fig S3

Fig S4

Fig S5

Fig S6

Fig S7

Table S1‐S13

## Data Availability

The data from present study are available in the Surveillance, Epidemiology, and End Results, https://seer.cancer.gov
