# Supplementary figures and images for: The role of surgery on the primary tumor site in bladder cancer with distant metastasis: significance of histology type and metastatic pattern
Source: Cancer Med. 2020 Oct 27;9(24):9293–302. doi: 10.1002/cam4.3560 (PMC7774714; doi:10.1002/cam4.3560)

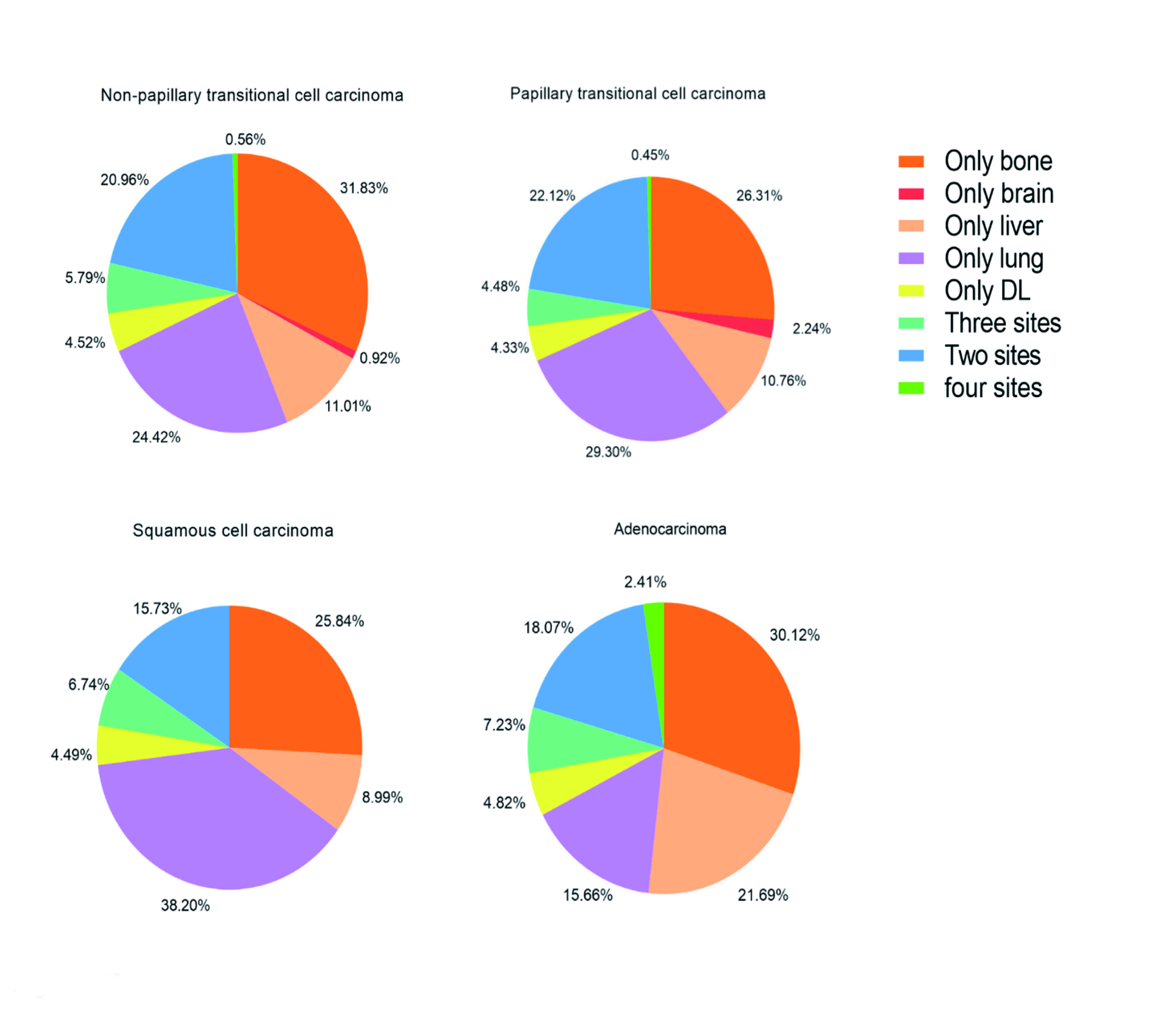

Supplement: Supplementary file 1 — Fig S1 [file CAM4-9-9293-s001.tif]

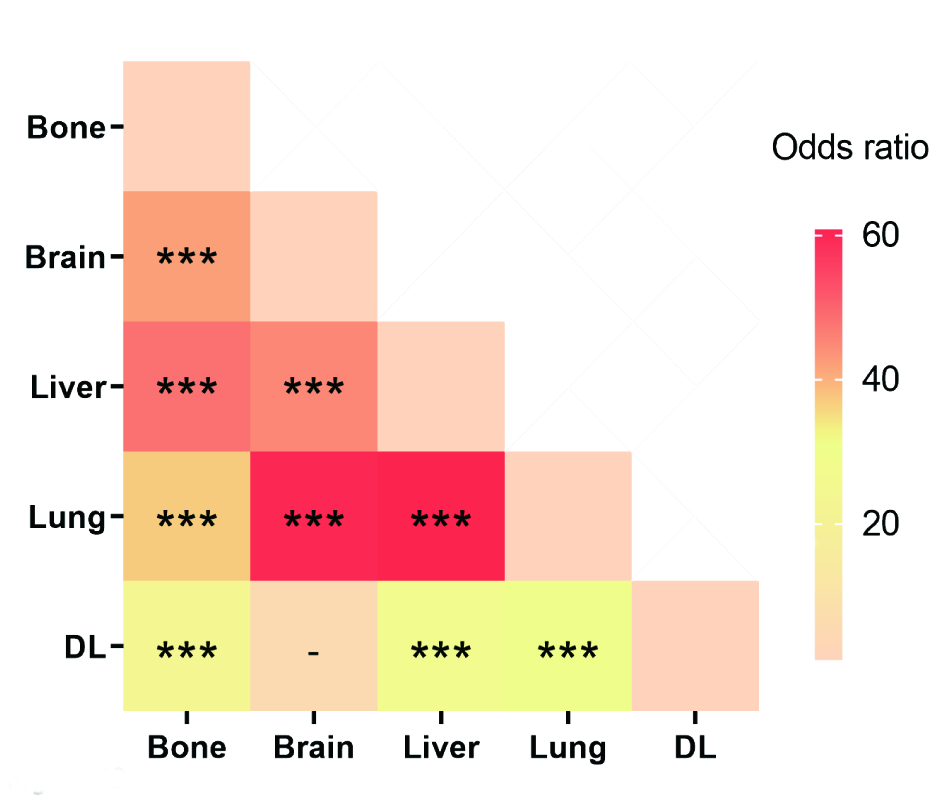

Supplement: Supplementary file 2 — Fig S2 [file CAM4-9-9293-s002.tif]

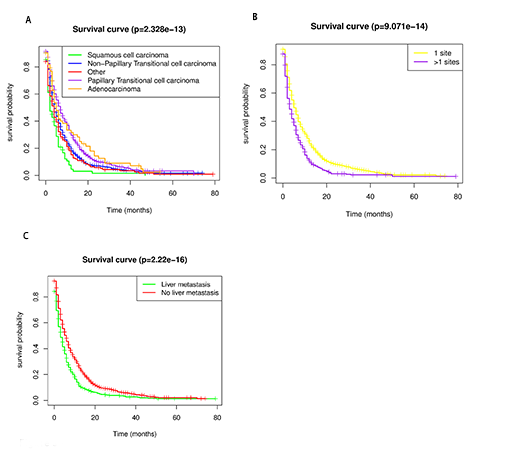

Supplement: Supplementary file 3 — Fig S3 [file CAM4-9-9293-s003.tif]

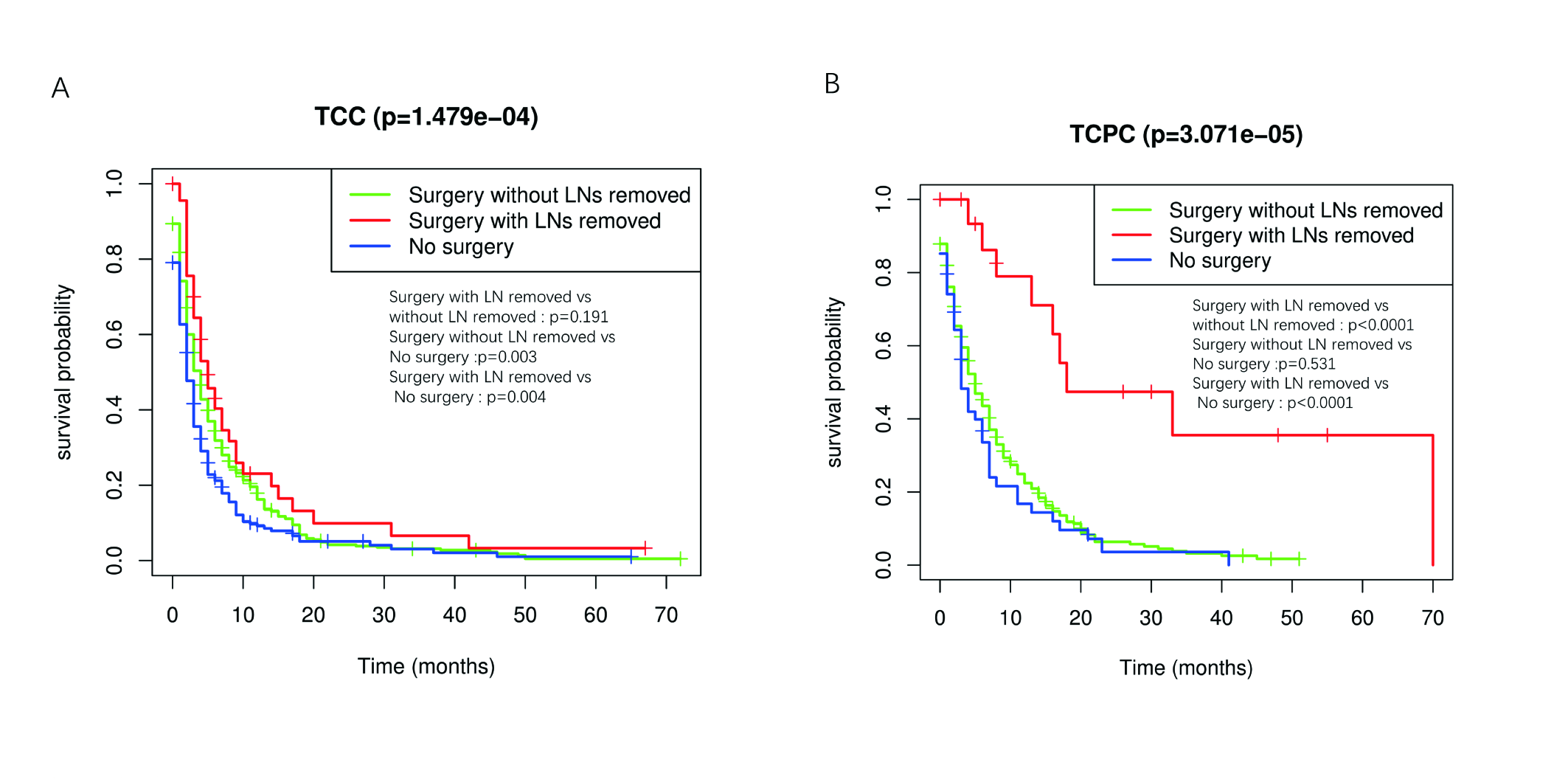

Supplement: Supplementary file 4 — Fig S4 [file CAM4-9-9293-s004.tif]

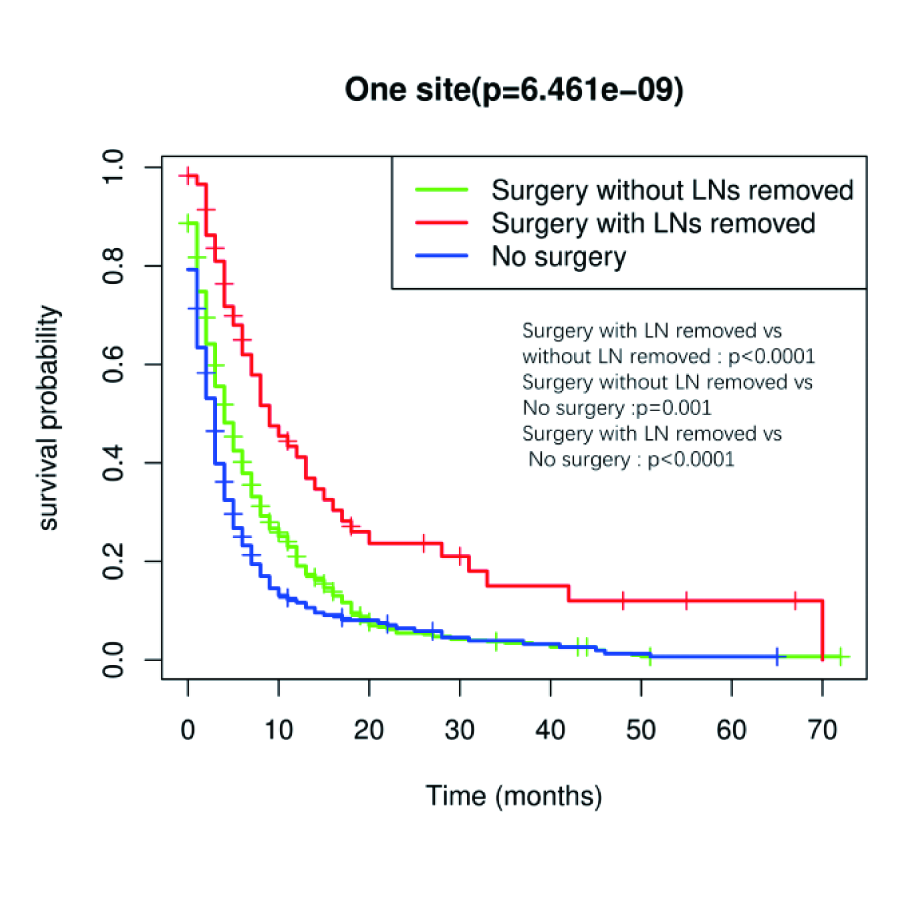

Supplement: Supplementary file 5 — Fig S5 [file CAM4-9-9293-s005.tif]

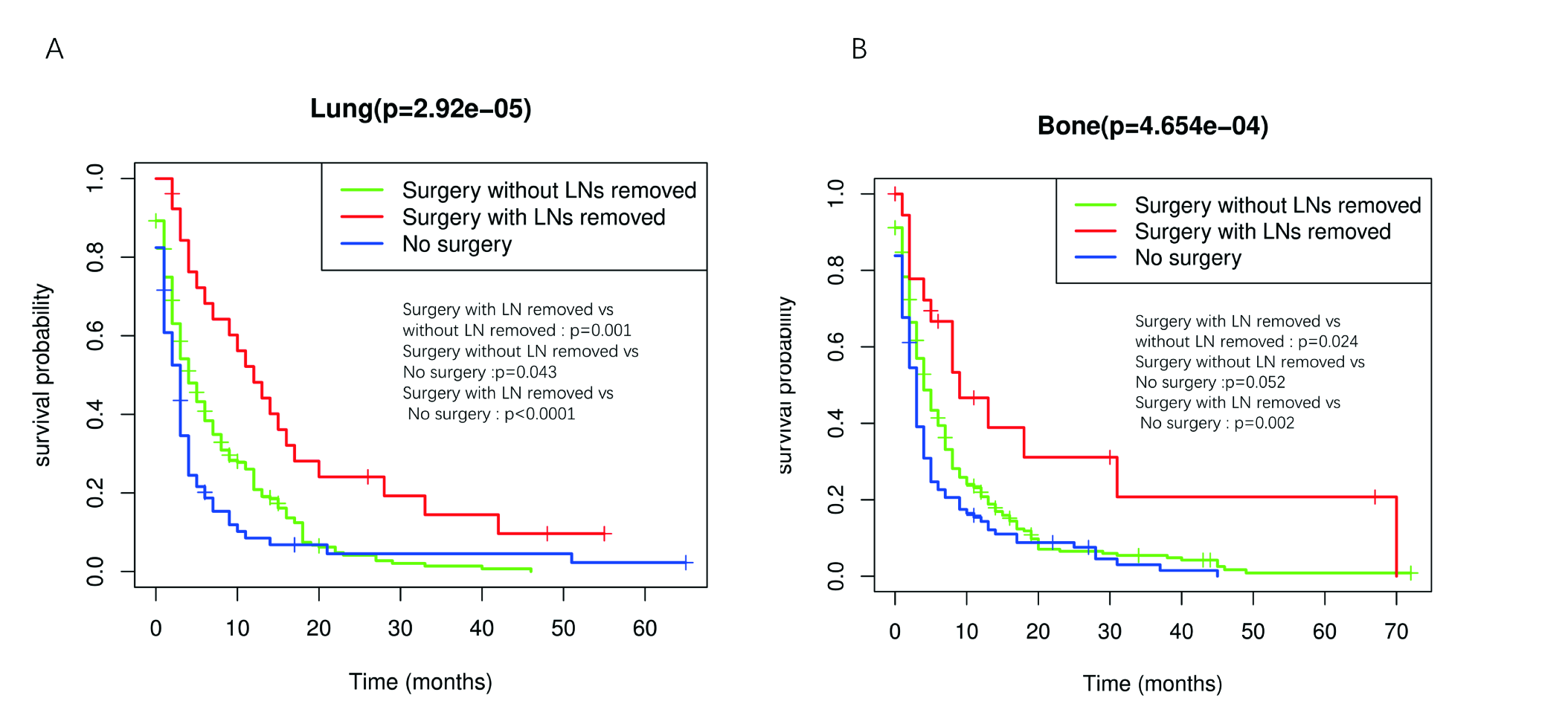

Supplement: Supplementary file 6 — Fig S6 [file CAM4-9-9293-s006.tif]

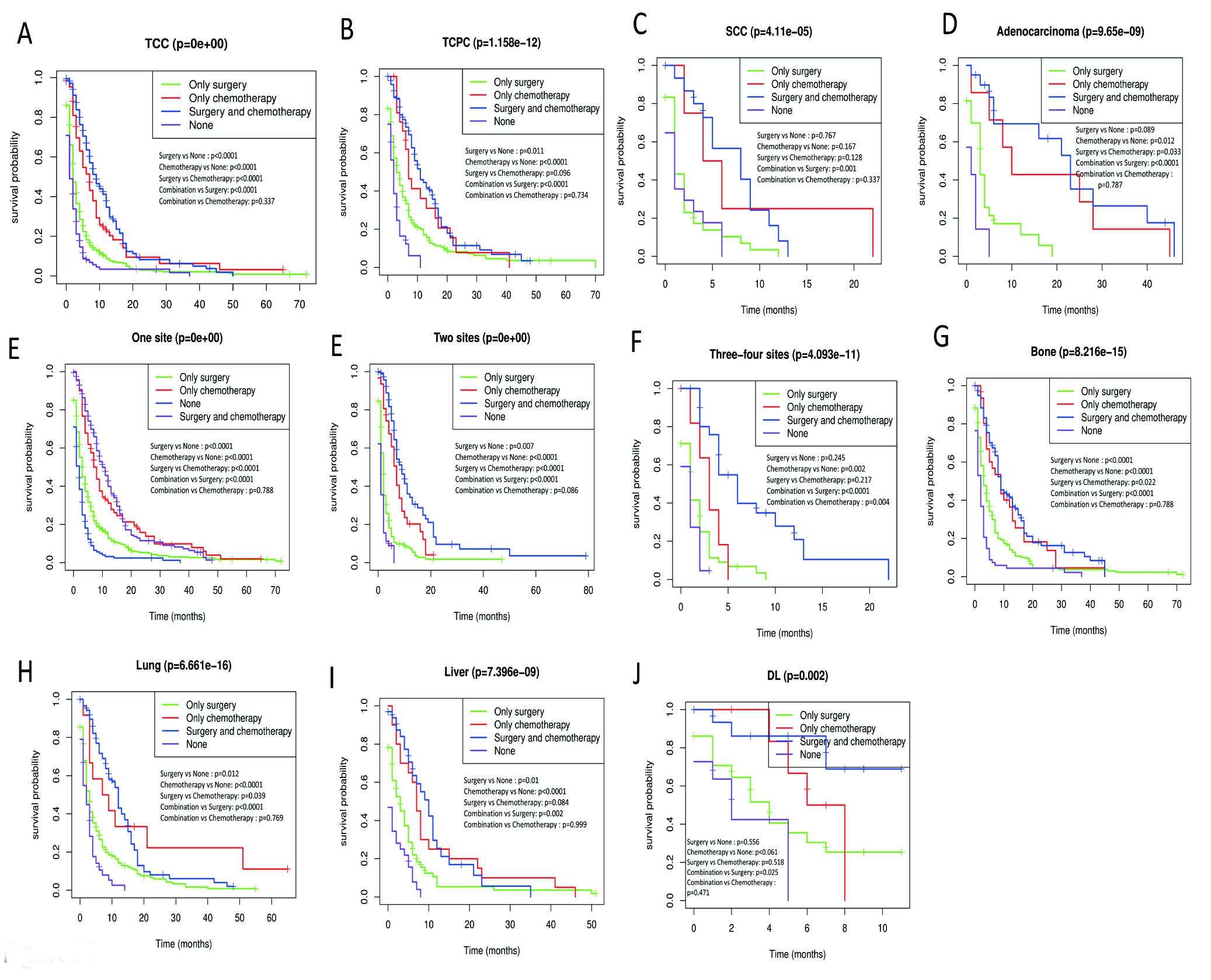

Supplement: Supplementary file 7 — Fig S7 [file CAM4-9-9293-s007.tif]
