# Supplementary material for: The role of surgery on the primary tumor site in bladder cancer with distant metastasis: significance of histology type and metastatic pattern
Source: Cancer Med. 2020 Oct 27;9(24):9293–302. doi: 10.1002/cam4.3560 (PMC7774714; doi:10.1002/cam4.3560)
Supplement: Supplementary file 8 — Table S1‐S13 [file CAM4-9-9293-s008.docx]

Supplementary Table1 Clinical characteristics comparison between bladder cancers with metastasis and without metastasis.

| Covariates | Total  (n=91744) | No-metastasis  (n=89274) | Metastasis  (n=2470) | P-value |
| --- | --- | --- | --- | --- |
| Age at diagnosis(years) |  |  |  | **0.0003** |
| <40 | 822(0.90%) | 805(0.90%) | 17(0.69%) |  |
| 40-49 | 2562(2.79%) | 2476(2.77%) | 86(3.48%) |  |
| 50-59 | 10961(11.95%) | 10609(11.88%) | 352(14.25%) |  |
| 60-69 | 23938(26.09%) | 23343(26.15%) | 595(24.09%) |  |
| 70-79 | 27972(30.49%) | 27199(30.47%) | 773(24.09%) |  |
| >=80 | 25489(27.78%) | 24842(27.83%) | 647(26.19%) |  |
| Race |  |  |  | **<0.0001** |
| White | 81885(89.25%) | 79815(89.40%) | 2070(83.81%) |  |
| Black | 5597(6.10%) | 5318(5.96%) | 279(11.30%) |  |
| Other | 4262(4.65%) | 4141(4.64%) | 2070(83.81%) |  |
| Sex |  |  |  | **<0.0001** |
| Female | 21881(23.85%) | 21191(23.74%) | 690(27.94%) |  |
| Male | 69863(76.15%) | 68083(76.26%) | 1780(27.94%) |  |
| Primary Site |  |  |  | **<0.0001** |
| Trigone of bladder | 6020(6.56%) | 5875(6.58%) | 145(5.87%) |  |
| Dome of bladder | 3395(3.70%) | 3328(3.73%) | 67(2.71%) |  |
| Lateral wall of bladder | 19549(21.31%) | 19254(21.57%) | 295(11.94%) |  |
| Anterior wall of bladder | 2170(2.37%) | 2099(2.35%) | 71(2.87%) |  |
| Posterior wall of bladder | 8454(9.21%) | 8299(9.30%) | 155(6.28%) |  |
| Bladder neck | 2865(3.12%) | 2761(3.09%) | 104(4.21%) |  |
| Ureteric orifice | 3186(3.47%) | 3141(3.52%) | 45(1.82%) |  |
| Urachus | 154(0.17%) | 144(0.16%) | 10(0.40%) |  |
| Overlapping lesion of bladder | 9963(10.86%) | 9517(10.66%) | 446(18.06%) |  |
| Bladder, NOS | 35988(39.23%) | 34856(39.04%) | 1132(45.83%) |  |
| Histologic type |  |  |  | **<0.0001** |
| Non-Papillary Transitional cell carcinoma | 24321(26.51%) | 22904(25.66%) | 1417(57.37%) |  |
| Papillary Transitional cell carcinoma | 63863(69.61%) | 63194(70.79%) | 669(27.09%) |  |
| Squamous cell neoplasms | 1437(1.57%) | 1348(1.51%) | 89(3.60%) |  |
| Adenomas and adenocarcinoma | 716(0.78%) | 633(0.71%） | 83(3.36%) |  |
| Other | 1407(1.53%) | 1195(1.34%) | 212(8.58%) |  |
| Grade |  |  |  | **<0.0001** |
| I | 11124(12.13%) | 11094(12.43%) | 30(1.21%) |  |
| II | 24324(26.51%) | 24223(27.13%) | 101(4.09%) |  |
| III | 14730(16.06%) | 14064(15.75%) | 666(26.96%) |  |
| IV | 41566(45.31%) | 39893(44.69%) | 1673(67.73%) |  |
| Regional nodes positives(number) |  |  |  | **<0.0001** |
| 0 | 7394(8.06%) | 7319(8.20%) | 75(3.04%) |  |
| 1-3 | 1744(8.06%) | 1689(1.89%) | 55(2.23%) |  |
| >3 | 809(1.90%) | 775(0.87%) | 34(1.38%) |  |
| other | 81797(89.16%) | 79491(89.04%) | 2306(93.3) |  |
| Radiotherapy |  |  |  | **<0.0001** |
| Refused | 281(0.31%) | 256(0.29%) | 25(1.01%) |  |
| Yes | 4923(5.37%) | 4372(4.90%) | 571(23.12%) |  |
| None/unknown | 86540(94.33%) | 84646(94.82%) | 1874(75.87%) |  |
| Chemotherapy |  |  |  | **<0.0001** |
| Yes | 25871(28.20%) | 24683(27.65%) | 1188(48.10%) |  |
| None/Unknown | 65873(71.80%) | 64591(72.35%) | 1282(51.90%) |  |
| Surgery |  |  |  | **<0.0001** |
| Yes | 87905(95.82%) | 85791(96.10%) | 2114(85.59%) |  |
| No | 3839(4.18%) | 3483(3.90%) | 356(14.41%) |  |

Supplementary Table2 Univariable and multivariable Cox regression model analyses of overall survival in bladder cancers.

| Variables | level | Univariable | | | multivariable | | |
| --- | --- | --- | --- | --- | --- | --- | --- |
|  |  | P | HR | 95%CI | P | HR | 95%CI |
| Age at diagnosis(years) | <40 | Ref |  |  |  |  |  |
|  | 40-49 | **0.0001** | 1.638 | 1.276-2.102 | **0.002** | 1.488 | 1.159-1.910 |
|  | 50-59 | **<0.0001** | 1.953 | 1.546-2.467 | **<0.0001** | 1.782 | 1.410-2.252 |
|  | 60-69 | **<0.0001** | 2.250 | 1.786-2.835 | **<0.0001** | 2.083 | 1.653-2.626 |
|  | 70-79 | **<0.0001** | 3.442 | 2.734-4.334 | **<0.0001** | 3.165 | 2.513-3.987 |
|  | >=80 | **<0.0001** | 6.985 | 5.549-8.792 | **<0.0001** | 6.307 | 5.008-7.943 |
| Race | White | Ref |  |  |  |  |  |
|  | Black | **<0.0001** | 1.363 | 1.304-1.425 | **<0.0001** | 1.320 | 1.262-1.381 |
|  | Other | **0.006** | 0.920 | 0.867-0.976 | **<0.0001** | 0.867 | 0.817-0.920 |
| Sex | Female | Ref |  |  |  |  |  |
|  | Male | **<0.0001** | 0.875 | 0.852-0.899 | **<0.0001** | 0.931 | 0.906-0.957 |
| Primary Site | Lateral wall of bladder | Ref |  |  |  |  |  |
|  | Dome of bladder | **<0.0001** | 1.401 | 1.313-1.494 | 0.333 | 1.032 | 0.968-1.101 |
|  | Trigone of bladder | **<0.0001** | 1.217 | 1.152-1.285 | **<0.0001** | 1.160 | 1.098-1.225 |
|  | Anterior wall of bladder | **<0.0001** | 1.578 | 1.463-1.703 | **0.003** | 1.121 | 1.039-1.210 |
|  | Posterior wall of bladder | **0.003** | 1.108 | 1.055-1.164 | 0.663 | 0.989 | 0.942-1.039 |
|  | Bladder neck | **<0.0001** | 1.432 | 1.338-1.534 | <0.0001 | 1.131 | 1.056-1.212 |
|  | Ureteric orifice | **<0.0001** | 0.821 | 0.759-0.888 | 0.904 | 0.995 | 0.920-1.077 |
|  | Ureteric orifice | 0.651 | 0.926 | 0.664-1.292 | 0.082 | 0.741 | 0.529-1.039 |
|  | Overlapping lesion of bladder | **9<0.0001** | 1.811 | 1.737-1.888 | <  **<0.0001** | 11.355 | 1.299-1.414 |
|  | Bladder, NOS | **0.003** | 1.313 | 1.270-1.358 | **<0.0001** | 1.146 | 1.108-1.185 |
| Histologic type | Non-papillary transitional cell carcinoma | Ref |  |  |  |  |  |
|  | Papillary Transitional cell carcinoma | **<0.0001** | 0.380 | 0.371-0.389 | **<0.0001** | 0.517 | 0.504-0.531 |
|  | Squamous cell neoplasms | **<0.0001** | 1.629 | 1.520-1.745 | **<0.0001** | 2.161 | 2.015-2.138 |
|  | Adenomas and adenocarcinoma | **0.033** | 1.121 | 1.009-1.244 | **<0.0001** | 1.290 | 1.160-1.435 |
|  | Other | **<0.0001** | 1.680 | 1.569-1.800 | **<0.0001** | 1.492 | 1.392-1.600 |
| Grade | I | Ref |  |  |  |  |  |
|  | II | **0.001** | 1.098 | 1.042-1.158 | **0.003** | 1.082 | 1.026-1.141 |
|  | III | **<0.0001** | 3.040 | 2.893-3.194 | **<0.0001** | 2.039 | 1.938-2.145 |
|  | IV | **<0.0001** | 2.889 | 2.759-3.025 | **<0.0001** | 2.025 | 1.931-2.124 |
| Regional nodes positive(number) | 0 | Ref |  |  |  |  |  |
|  | 1-3 | **<0.0001** | 2.690 | 2.500-2.894 | **<0.0001** | 2.436 | 2.264-2.621 |
|  | >3 | **<0.0001** | 3.547 | 3.231-3.893 | **<0.0001** | 3.192 | 2.907-3.505 |
|  | other | 0.228 | 1.027 | 0.983-1.073 | **<0.0001** | 1.356 | 1.296-1.419 |
| Metastasis | No | Ref |  |  |  |  |  |
|  | yes | **<0.0001** | 9.014 | 8.609-9.437 | **<0.0001** | 5.714 | 5.448-5.992 |

Supplementary Table3 Univariable and multivariable Cox regression model analyses of overall survival in Non-papillary distant metastatic bladder cancers after PSM

| Variables | level |  |  |  |  |  |  |
| --- | --- | --- | --- | --- | --- | --- | --- |
|  |  | P | HR | 95%CI | P | HR | 95%CI |
| Age at diagnosis(years) | <70 | Ref |  |  |  |  |  |
|  | >=70 | 0.082 | 1.149 | 0.983-1.343 |  |  |  |
| Race | White | Ref |  |  |  |  |  |
|  | Black | 0.268 | 1.138 | 0.905-1.431 |  |  |  |
|  | Other | 0.223 | 0.786 | 0.534-1.157 |  |  |  |
| Sex | Female | Ref |  |  |  |  |  |
|  | Male | 0.095 | 0.868 | 0.736-1.025 |  |  |  |
| Primary Site | Trigone of bladder | Ref |  |  |  |  |  |
|  | Dome of bladder | 0.642 | 1.157 | 0.626-2.138 |  |  |  |
|  | Lateral wall of bladder | 0.827 | 0.950 | 0.600-1.505 |  |  |  |
|  | Anterior wall of bladder | 0.833 | 0.935 | 0.498-1.735 |  |  |  |
|  | Posterior wall of bladder | 0.199 | 1.377 | 0.845-2.244 |  |  |  |
|  | Bladder neck | 0.357 | 0.782 | 0.463-1.321 |  |  |  |
|  | Ureteric orifice | 0.128 | 0.586 | 0.295-1.165 |  |  |  |
|  | Overlapping lesion of bladder | 0.739 | 0.936 | 0.633-1.384 |  |  |  |
|  | Bladder, NOS | 0.774 | 0.948 | 0.661-1.361 |  |  |  |
| Grade | II | Ref |  |  |  |  |  |
|  | III | 0.078 | 1.498 | 0.956-2.345 | 0.053 | 1.559 | 0.993-2.445 |
|  | IV | **0.040** | 1.586 | 1.022-2.462 | **0.019** | 1.695 | 1.089-2.636 |
| T stage | T0 | Ref |  |  |  |  |  |
|  | T1 | 0.233 | 3.323 | 0.461-23.947 |  |  |  |
|  | T2 | 0.205 | .568 | 0.499-25.513 |  |  |  |
|  | T3 | 0.293 | 2.893 | 0.400-20.941 |  |  |  |
|  | T4 | 0.221 | 3.416 | 0.477-24.450 |  |  |  |
|  | TX + unknown | 0.221 | 3.420 | 0.478-24.450 |  |  |  |
| N stage | N0 | Ref |  |  |  |  |  |
|  | N1 | 0.508 | 1.101 | 0.828-1.464 |  |  |  |
|  | N2 | 0.616 | 0.944 | 0.754-1.183 |  |  |  |
|  | N3 | 0.221 | 1.257 | 0.871-1.813 |  |  |  |
|  | NX + unknown | 0.534 | 0.942 | 0.781-1.137 |  |  |  |
| Surgery | No | Ref |  |  |  |  |  |
|  | Yes | **<0.0001** | 0.740 | 0.626-0.875 | **<0.0001** | 0.680 | 0.574-0.805 |
| Surgery about regional lymph nodes | Surgery but no LNs removed | Ref |  |  |  |  |  |
|  | No surgery | **<0.001** | 1.317 | 1.112-1.560 |  |  |  |
|  | Surgery and LN removed | 0.105 | 0.760 | 0.545-1.060 |  |  |  |
| Radiotherapy | Refused | Ref |  |  |  |  |  |
|  | Yes | 0.134 | 0.534 | 0.236-1.212 |  |  |  |
|  | None/Unknown | 0.232 | 0.612 | 0.273-1.370 |  |  |  |
| Radiation sequence with surgery | No radiotherapy and/or surgery | Ref |  |  |  |  |  |
|  | Radiation after surgery | 0.097 | 0.829 | 0.664-1.035 |  |  |  |
|  | Radiation before surgery | 0.122 | 0.407 | 0.131-1.270 |  |  |  |
|  | other | 0.267 | 0.525 | 00.169-1.636 |  |  |  |
| Chemotherapy | No/Unknown | Ref |  |  |  |  |  |
|  | Yes | **<0.0001** | 0.405 | 0.340-0.482 | **<0.0001** | 0.369 | 0.309-0.441 |
| Number of sites of metastases | 1 | Ref |  |  |  |  |  |
|  | 2 | 0.194 | 1.130 | 0.940-1.360 | **0.043** | 1.223 | 1.006-1.485 |
|  | 3 | **0.002** | 1.617 | 1.202-2.177 | **0.009** | 1.551 | 1.118-2.150 |
|  | 4 | 0.570 | 1.242 | 0.588-2.621 | 0.202 | 1.649 | 0.764-3.556 |
| Metastases sites including liver | No | Ref |  |  |  |  |  |
|  | Yes | **<0.0001** | 1.453 | 1.229-1.717 | **0.008** | 1.649 | 0.764-1.559 |
| Metastases sites including brain | No | Ref |  |  |  |  |  |
|  | Yes | 0.622 | 0.908 | 0.617-1.334 |  |  |  |

Supplementary Table4 Univariable and multivariable Cox regression model analyses of overall survival in papillary distant metastatic bladder cancers after PSM

| Variables | level |  |  |  |  |  |  |
| --- | --- | --- | --- | --- | --- | --- | --- |
|  |  | P | HR | 95%CI | P | HR | 95%CI |
| Age at diagnosis(years) | <70 | Ref |  |  |  |  |  |
|  | >=70 | **0.003** | 1.437 | 1.133-1.823 | **0.030** | 1.308 | 1.026-1.668 |
| Race | White | Ref |  |  |  |  |  |
|  | Black | 0.346 | 0.841 | 0.587-1.205 |  |  |  |
|  | Other | 0.377 | 1.250 | 0.762-2.048 |  |  |  |
| Sex | Female | Ref |  |  |  |  |  |
|  | Male | 0.526 | 0.917 | 0.703-1.198 |  |  |  |
| Primary Site | Trigone of bladder | Ref |  |  |  |  |  |
|  | Dome of bladder | 0.087 | 0.332 | 0.094-1.171 |  |  |  |
|  | Lateral wall of bladder | 0.986 | 1.006 | 0.509-1.988 |  |  |  |
|  | Anterior wall of bladder | 0.186 | 0.497 | 0.176-1.401 |  |  |  |
|  | Posterior wall of bladder | 0.434 | 1.369 | 0.624-3.004 |  |  |  |
|  | Bladder neck | 0.702 | 0.847 | 0.362-1.998 |  |  |  |
|  | Ureteric orifice | 0.734 | 1.182 | 0.449-3.114 |  |  |  |
|  | Overlapping lesion of bladder | 0.740 | 0.905 | 0.501-1.635 |  |  |  |
|  | Bladder, NOS | 0.372 | 0.772 | 0.437-1.362 |  |  |  |
| Grade | II | Ref |  |  |  |  |  |
|  | III | 0.280 | 0.809 | 0.550-1.189 | 0.996 | 0.999 | 0.673-1.484 |
|  | IV | **0.032** | 0.683 | 0.482-0.967 | 0.160 | 0.775 | 0.543-1.106 |
| T stage | T1 | Ref |  |  |  |  |  |
|  | T2 | 0.058 | 1.353 | 0.990-1.849 |  |  |  |
|  | T3 | 0.916 | 1.034 | 0.556-1.920 |  |  |  |
|  | T4 | 0.947 | 0.987 | 0.676-1.443 |  |  |  |
|  | TX + unknown | 0.169 | 1.278 | 0.901-1.814 |  |  |  |
| N stage | N0 | Ref |  |  |  |  |  |
|  | N1 | 0.498 | 1.151 | 0.767-1.726 |  |  |  |
|  | N2 | 0.970 | 1.007 | 0.697-1.455 |  |  |  |
|  | N3 | 0.430 | 1.311 | 0.669-2.572 |  |  |  |
|  | NX + unknown | 0.796 | 1.040 | 0.774-1.396 |  |  |  |
| Surgery | No | Ref |  |  |  |  |  |
|  | Yes | 0.110 | 1.290 | 0.944-1.764 |  |  |  |
| Surgery about regional lymph nodes | Surgery but no LNs removed | Ref |  |  |  |  |  |
|  | No surgery | 0.290 | 1.184 | 0.866-1.620 |  |  |  |
|  | Surgery and LN removed | **<0.0001** | 0.251 | 0.123-0.511 | **0.001** | 0.292 | 0.141-0.602 |
| Radiotherapy | Refused | Ref |  |  |  |  |  |
|  | Yes | 0.794 | 1.167 | 0.367-3.714 |  |  |  |
|  | None/Unknown | 0.915 | 0.940 | 0.300-2.944 |  |  |  |
| Radiation sequence with surgery | No radiotherapy and/or surgery | Ref |  |  |  |  |  |
|  | Radiation after surgery | 0.246 | 1.198 | 0.883-1.627 |  |  |  |
|  | Radiation before surgery | 0.582 | 1.738 | 0.243-12.430 |  |  |  |
|  | other | 0.203 | 2.099 | 0.670-6.579 |  |  |  |
| Chemotherapy | No/Unknown | Ref |  |  |  |  |  |
|  | Yes | **<0.0001** | 0.473 | 0.366-0.610 | **<0.0001** | 0.441 | 0.336-0.579 |
| Number of sites of metastases | 1 | Ref |  |  |  |  |  |
|  | 2 | **0.024** | 1.362 | 1.042-1.781 | **0.024** | 1.378 | 1.043-1.819 |
|  | 3-4 | **<0.0001** | 2.821 | 1.759-4.525 | **0.023** | 2.059 | 1.105-3.838 |
| Metastases sites including liver | No | Ref |  |  |  |  |  |
|  | Yes | **<0.0001** | 1.724 | 1.349-2.204 | **0.007** | 1.436 | 1.102-1.870 |
| Metastases sites including brain | No | Ref |  |  |  |  |  |
|  | Yes | **0.024** | 2.012 | 1.094-3.699 | 0.876 | 1.064 | 0.488-2.320 |

Supplementary Table5 Univariable and multivariable Cox regression model analyses of overall survival in squamous cell bladder cancers after PSM

| Variables | level |  |  |  |  |  |  |
| --- | --- | --- | --- | --- | --- | --- | --- |
|  |  | P | HR | 95%CI | P | HR | 95%CI |
| Age at diagnosis(years) | <70 |  |  |  |  |  |  |
|  | >=70 | **0.007** | 1.994 | 1.208-3.291 | 0.242 | 1.378 | 0.805-2.361 |
| Race | White | Ref |  |  |  |  |  |
|  | Black | 0.411 | 1.278 | 0.712-2.293 |  |  |  |
|  | Other | 0.465 | 1.702 | 0.408-7.097 |  |  |  |
| Sex | Female | Ref |  |  |  |  |  |
|  | Male | 0.262 | 0.754 | 0.461-1.235 |  |  |  |
| Primary Site | Trigone of bladder | Ref |  |  |  |  |  |
|  | Lateral wall of bladder | 0.403 | 2.034 | 0.386-10.733 |  |  |  |
|  | Anterior wall of bladder | 0.338 | 3.278 | 0.289-37.183 |  |  |  |
|  | Posterior wall of bladder | 0.786 | 1.257 | 0.241-6.565 |  |  |  |
|  | Bladder neck | 0.923 | 0.908 | 0.126-6.519 |  |  |  |
|  | Overlapping lesion of bladder | 0.588 | 1.531 | 0.328-7.136 |  |  |  |
|  | Bladder, NOS | 0.440 | 1.754 | 0.421-7.312 |  |  |  |
| Grade | II | Ref |  |  |  |  |  |
|  | III | 0.972 | 1.010 | 0.594-1.717 |  |  |  |
|  | IV | 0.757 | 1.115 | 0.558-2.228 |  |  |  |
| T stage | T1 | Ref |  |  |  |  |  |
|  | T2 | 0.788 | 0.892 | 0.390-2.043 |  |  |  |
|  | T3 | 0.154 | 0.453 | 0.152-1.345 |  |  |  |
|  | T4 | 0.167 | 0.582 | 0.270-1.254 |  |  |  |
|  | TX + unknown | 0.163 | 0.574 | 0.263-1.253 |  |  |  |
| N stage | N0 | Ref |  |  |  |  |  |
|  | N1 | **0.029** | 0.892 | 0.390-2.043 |  |  |  |
|  | N2 | 0.154 | 0.453 | 0.152-1.345 |  |  |  |
|  | N3 | 0.167 | 0.582 | 0.270-1.254 |  |  |  |
|  | NX + unknown | 0.163 | 0.574 | 0.263-1.253 |  |  |  |
| Surgery | No | Ref |  |  |  |  |  |
|  | Yes | 0.463 | 0.820 | 0.484-1.392 | 0.556 | 0.854 | 0.499-1.462 |
| Surgery about regional lymph nodes | Surgery but no LNs removed | Ref |  |  |  |  |  |
|  | No surgery | 0.982 | 0.994 | 0.570-1.732 |  |  |  |
|  | Surgery and LN removed | 0.053 | 0.500 | 0.248-1.009 |  |  |  |
| Radiotherapy | Refused | Ref |  |  |  |  |  |
|  | Yes | 0.577 | 0.553 | 0.069-4.427 |  |  |  |
|  | None/Unknown | 0.822 | 0.797 | 0.109-5.811 |  |  |  |
| Radiation sequence with surgery | No radiotherapy and/or surgery | Ref |  |  |  |  |  |
|  | Radiation after surgery | 0.131 | 0.457 | 0.165-1.263 |  |  |  |
| Chemotherapy | No/Unknown | Ref |  |  |  |  |  |
|  | Yes | **<0.0001** | 0.318 | 0.174-0.582 | **0.003** | 0.371 | 0.193-0.714 |
| Number of sites of metastases | 1 | Ref |  |  |  |  |  |
|  | 2 | 0.905 | 1.047 | 0.492-2.227 |  |  |  |
|  | 3 | 0.356 | 1.544 | 0.613-3.889 |  |  |  |
| Metastases sites including liver | No | Ref |  |  |  |  |  |
|  | Yes | 0.664 | 0.882 | 0.501-1.554 |  |  |  |

Supplementary Table6 Univariable and multivariable Cox regression model analyses of overall survival in adenocarcinoma bladder cancers after PSM

| Variables | level |  |  |  |  |  |  |
| --- | --- | --- | --- | --- | --- | --- | --- |
|  |  | P | HR | 95%CI | P | HR | 95%CI |
| Age at diagnosis(years) | <70 | Ref |  |  |  |  |  |
|  | >=70 | **0.029** | 1.917 | 1.070-3.434 | 0.748 | 0.892 | 0.446-1.786 |
| Race | White | Ref |  |  |  |  |  |
|  | Black | 0.192 | 1.681 | 0.771-3.668 |  |  |  |
|  | Other | 0.177 | 1.842 | 0.758-4.473 |  |  |  |
| Sex | Female | Ref |  |  |  |  |  |
|  | Male | 0.735 | 0.898 | 0.481-1.675 |  |  |  |
| Primary Site | Trigone of bladder | Ref |  |  |  |  |  |
|  | Dome of bladder | 0.072 | 0.159 | 0.021-1.181 |  |  |  |
|  | Lateral wall of bladder | 0.781 | 0.789 | 0.149-4.168 |  |  |  |
|  | Anterior wall of bladder | 0.194 | 0.264 | 0.035-1.970 |  |  |  |
|  | Posterior wall of bladder | 0.246 | 0.356 | 0.062-2.037 |  |  |  |
|  | Bladder neck | 0.392 | 0.452 | 0.074-2.780 |  |  |  |
|  | Urachus | **0.033** | 0.071 | 0.006-0.807 |  |  |  |
|  | Overlapping lesion of bladder | 0.508 | 0.596 | 0.1128-2.763 |  |  |  |
|  | Bladder, NOS | 0.501 | 0.599 | 0.134-2.668 |  |  |  |
| Grade | II | Ref |  |  |  |  |  |
|  | III | **0.042** | 2.264 | 1.030-4.976 | 0.153 | 1.892 | 0.788-4.539 |
|  | IV | 0.093 | 2.144 | 0.879-5.556 | 0.198 | 1.865 | 0.721-4.822 |
| T stage | T0-TI | Ref |  |  |  |  |  |
|  | T2 | 0.233 | 0.564 | 0.220-1.415 |  |  |  |
|  | T3 | 0.479 | 0.611 | 0.156-2.388 |  |  |  |
|  | T4 | 0.371 | 0.649 | 0.252-1.675 |  |  |  |
|  | TX + unknown | 0.712 | 0.847 | 0.350-2.049 |  |  |  |
| N stage | N0 | Ref |  |  |  |  |  |
|  | N1 | 0.272 | 1.813 | 0.627-5.247 |  |  |  |
|  | N2 | 0.323 | 1.564 | 0.645-3.796 |  |  |  |
|  | NX + unknown | **0.029** | 2.314 | 1.090-4.915 |  |  |  |
| Surgery | No |  |  |  |  |  |  |
|  | Yes | 0.415 | 0.770 | 0.411-1.443 | 0.056 | 0.451 | 0.199-1.021 |
| Surgery about regional lymph nodes | Surgery but no LNs removed | Ref |  |  |  |  |  |
|  | No surgery | 0.534 | 1.223 | 0.649-2.307 |  |  |  |
|  | Surgery and LN removed | 0.334 | 0.493 | 0.118-2.069 |  |  |  |
| Radiotherapy | Refused | Ref |  |  |  |  |  |
|  | Yes | 0.100 | 0.454 | 0.177-1.165 |  |  |  |
|  | None/Unknown | 0.472 | 0.598 | .0.139-2.496 |  |  |  |
| Radiation sequence with surgery | No radiotherapy and/or surgery | Ref |  |  |  |  |  |
|  | Radiation after surgery | 0.100 | 0.454 | 0.177-1.165 |  |  |  |
|  | Radiation before surgery | 0.472 | 0.589 | 0.139-2.496 |  |  |  |
| Chemotherapy | No/Unknown | Ref |  |  |  |  |  |
|  | Yes | **<0.0001** | 0.170 | 0.081-0.359 | **<0.0001** | 0.085 | 0.033-0.216 |
| Number of sites of metastases | 1 | Ref |  |  |  |  |  |
|  | 2 | 0.283 | 1.499 | 0.716-3.139 | **0.020** | 2.766 | 1.176-6.506 |
|  | 3-4 | **0.020** | 2.989 | 1.185-7.540 | 0.537 | 1.456 | 0.442-4.800 |
| Metastases sites including liver | No | Ref |  |  |  |  |  |
|  | Yes | **0.026** | 1.985 | 1.086-3.628 | **0.024** | 2.416 | 1.123-5.202 |

Supplementary Table7 Univariable and multivariable Cox regression model analyses of overall survival in bladder cancers with one site metastasis after PSM

| Variables | level |  |  |  |  |  |  |
| --- | --- | --- | --- | --- | --- | --- | --- |
|  |  | P | HR | 95%CI | P | HR | 95%CI |
| Age at diagnosis(years) | <70 | Ref |  |  |  |  |  |
|  | >=70 | **<0.0001** | 1.354 | 1.177-1.558 | **0.034** | 1.169 | 1.012-1.350 |
| Race | White | Ref |  |  |  |  |  |
|  | Black | 0.329 | 1.103 | 0.906-1.343 |  |  |  |
|  | Other | 0.540 | 0.899 | 0.640-1.263 |  |  |  |
| Sex | Female | Ref |  |  |  |  |  |
|  | Male | **0.009** | 0.821 | 0.709-0.951 | 0.656 | 0.966 | 0.831-1.124 |
| Primary Site | Trigone of bladder | Ref |  |  |  |  |  |
|  | Dome of bladder | 0.183 | 0.684 | 0.392-1.196 |  |  |  |
|  | Lateral wall of bladder | 0.733 | 0.931 | 0.617-1.404 |  |  |  |
|  | Anterior wall of bladder | 0.233 | 0.708 | 0.401-1.249 |  |  |  |
|  | Posterior wall of bladder | 0.252 | 1.281 | 0.838-1.958 |  |  |  |
|  | Bladder neck | 0.352 | 0.800 | 0.500-1.280 |  |  |  |
|  | Ureteric orifice | 0.291 | 0.677 | 0.328-1.398 |  |  |  |
|  | Urachus | **0.021** | 0.296 | 0.106-0.829 |  |  |  |
|  | Overlapping lesion of bladder | 0.895 | 1.024 | 0.724-1.448 |  |  |  |
|  | Bladder, NOS | 0.895 | 0.978 | 0.708-1.353 |  |  |  |
| Histologic type | Non-Papillary Transitional cell carcinoma | Ref |  |  |  |  |  |
|  | Papillary Transitional cell carcinoma | **<0.0001** | 0.721 | 0.610-0.852 | **0.001** | 0.757 | 0.639-0.897 |
|  | Squamous cell neoplasms | **0.004** | 1.518 | 1.146-2.009 | **0.001** | 1.626 | 1.221—2.166 |
|  | Adenomas and adenocarcinoma | **0.006** | 0.614 | 0.432-0.872 | 0.119 | 0.755 | 0.531-1.075 |
|  | Other | **0.018** | 1.319 | 1.050-1.657 | 0.052 | 1.258 | 0.998-1.586 |
| Grade | II | Ref |  |  |  |  |  |
|  | III | 0.185 | 1.185 | 0.922-1.524 |  |  |  |
|  | IV | 0.690 | 1.051 | 0.824-1.341 |  |  |  |
| T stage | T0 | Ref |  |  |  |  |  |
|  | T1 | 0.202 | 2.112 | 0.671-6.652 |  |  |  |
|  | T2 | 0.153 | 2.299 | 0.734-7.198 |  |  |  |
|  | T3 | 0.281 | 1.898 | 0.592-6.079 |  |  |  |
|  | T4 | 0.166 | 2.242 | 0.715-7.031 |  |  |  |
|  | TX + unknown | 0.151 | 2.309 | 0.736-7.244 |  |  |  |
| N stage | N0 | Ref |  |  |  |  |  |
|  | N1 | 0.431 | 1.109 | 0.857-1.436 |  |  |  |
|  | N2 | 0.774 | 0.970 | 0.789-1.193 |  |  |  |
|  | N3 | 0.735 | 1.063 | 0.746-1.516 |  |  |  |
|  | NX + unknown | 0.987 | 0.999 | 0.843-1.183 |  |  |  |
| Surgery | No | Ref |  |  |  |  |  |
|  | Yes | **<0.0001** | 0.718 | 0.616-0.837 | **<0.0001** | 0.729 | 0.622-0.855 |
| Surgery about regional lymph nodes | Surgery but no LNs removed | Ref |  |  |  |  |  |
|  | No surgery | **0.001** | 1.311 | 1.123-1.530 |  |  |  |
|  | Surgery and LN removed | **<0.0001** | 0.535 | 0.393-0.729 | **0.002** | 0.615 | 0.450-0.841 |
| Radiotherapy | Refused |  |  |  |  |  |  |
|  | Yes | 0.515 | 0.809 | 0.427-1.533 |  |  |  |
|  | None/Unknown | 0.635 | 0.860 | 0.460-1.606 |  |  |  |
| Radiation sequence with surgery | No radiotherapy and/or surgery | Ref |  |  |  |  |  |
|  | Radiation after surgery | 0.205 | 0.879 | 0.719-1.073 |  |  |  |
|  | Radiation before surgery | 0.102 | 0.440 | 0.164-1.177 |  |  |  |
| Chemotherapy | No/Unknown |  |  |  |  |  |  |
|  | Yes | **<0.0001** | 0.437 | 0.374-0.510 | **<0.0001** | 0.444 | 0.378-0.522 |
| Metastases sites including liver | No | Ref |  |  |  |  |  |
|  | Yes | **0.042** | 1.194 | 1.006-1.417 | 0.160 | 1.133 | 0.952-1.348 |
| Metastases sites including brain | No | Ref |  |  |  |  |  |
|  | Yes | 0.440 | 0.760 | 0.378-1.526 |  |  |  |

Supplementary Table8 Univariable and multivariable Cox regression model analyses of overall survival in bladder cancers with two sites metastasis after PSM

| Variables | level |  |  |  |  |  |  |
| --- | --- | --- | --- | --- | --- | --- | --- |
|  |  | P | HR | 95%CI | P | HR | 95%CI |
| Age at diagnosis(years) | <70 | Ref |  |  |  |  |  |
|  | >=70 | 0.100 | 1.219 | 0.963-1.542 |  |  |  |
| Race | White | Ref |  |  |  |  |  |
|  | Black | 0.857 | 0.970 | 0.694-1.355 |  |  |  |
|  | Other | 0.830 | 0.946 | 0.569-1.572 |  |  |  |
| Sex | Female | Ref |  |  |  |  |  |
|  | Male | 0.197 | 0.845 | 0.654-1.092 |  |  |  |
| Primary Site | Trigone of bladder | Ref |  |  |  |  |  |
|  | Dome of bladder | 0.524 | 0.709 | 0.246-2.044 |  |  |  |
|  | Lateral wall of bladder | 0.653 | 1.171 | 0.572-2.439 |  |  |  |
|  | Anterior wall of bladder | 0.973 | 0.982 | 0.341-2.830 |  |  |  |
|  | Posterior wall of bladder | 0.861 | 0.929 | 0.408-2.117 |  |  |  |
|  | Bladder neck | 0.515 | 1.340 | 0.555-3.236 |  |  |  |
|  | Ureteric orifice | 0.950 | 0.970 | 0.375-2.508 |  |  |  |
|  | Urachus | 0.647 | 0.620 | 0.080-4.808 |  |  |  |
|  | Overlapping lesion of bladder | 0.607 | 0.843 | 0.440-1.615 |  |  |  |
|  | Bladder, NOS | 0.879 | 0.953 | 0.517-1.758 |  |  |  |
| Histologic type | Non-Papillary Transitional cell carcinoma | Ref |  |  |  |  |  |
|  | Papillary Transitional cell carcinoma | 0.337 | 0.872 | 0.659-1.153 |  |  |  |
|  | Squamous cell neoplasms | 0.526 | 1.259 | 0.617-2.571 |  |  |  |
|  | Adenomas and adenocarcinoma | 0.420 | 0.768 | 0.405-1.458 |  |  |  |
|  | Other | 0.575 | 0.903 | 0.631-1.291 |  |  |  |
| Grade | II | Ref |  |  |  |  |  |
|  | III | 0.674 | 1.118 | 0.665-1.878 |  |  |  |
|  | IV | 0.864 | 1.045 | 0.634-1.721 |  |  |  |
| T stage | T0 | Ref |  |  |  |  |  |
|  | T1 | 0.516 | 0.517 | 0.070-3.796 |  |  |  |
|  | T2 | 0.550 | 0.547 | 0.076-3.950 |  |  |  |
|  | T3 | 0.606 | 0.590 | 0.079-4.389 |  |  |  |
|  | T4 | 0.544 | 0.541 | 0.075-3.928 |  |  |  |
|  | TX + unknown | 0.555 | 0.552 | 0.077-3.967 |  |  |  |
| N stage | N0 | Ref |  |  |  |  |  |
|  | N1 | 0.189 | 0.748 | 0.486-1.153 |  |  |  |
|  | N2 | 0.955 | 1.010 | 0.7028-1.442 |  |  |  |
|  | N3 | 0.186 | 1.444 | 0.837-2.489 |  |  |  |
|  | NX + unknown | 0.912 | 1.017 | 0.761-1.358 |  |  |  |
| Surgery | No | Ref |  |  |  |  |  |
|  | Yes | **0.018** | 0.722 | 0.551-0.947 | **<0.0001** | 1.558 | 1.222-1.985 |
| Surgery about regional lymph nodes | Surgery but no LNs removed | Ref |  |  |  |  |  |
|  | No surgery | **0.022** | 1.374 | 1.046-1.805 |  |  |  |
|  | Surgery and LN removed | 0.678 | 0.884 | 0.493-1.584 |  |  |  |
| Radiotherapy | Refused | Ref |  |  |  |  |  |
|  | Yes | 0.113 | 0.319 | 0.078-1.311 |  |  |  |
|  | None/Unknown | 0.131 | 0.341 | 0.084-1.377 |  |  |  |
| Radiation sequence with surgery | No radiotherapy and/or surgery | Ref |  |  |  |  |  |
|  | Radiation after surgery | 0.865 | 1.030 | 0.735-1.442 |  |  |  |
|  | Radiation before surgery | 0.708 | 1.305 | 0.324-5.264 |  |  |  |
|  | other | 0.202 | 0.475 | 0.151-1.489 |  |  |  |
| Chemotherapy | No/Unknown | Ref |  |  |  |  |  |
|  | Yes | **<0.0001** | 0.284 | 0.218-0.371 | **<0.0001** | 0.255 | 0.194-0.335 |
| Metastases sites including liver | No | Ref |  |  |  |  |  |
|  | Yes | **0.002** | 1.470 | 1.158-1.866 | **<0.0001** | 0.589 | 0.447-0.777 |
| Metastases sites including brain | No | Ref |  |  |  |  |  |
|  | Yes | 0.830 | 1.059 | 0.628-1.784 |  |  |  |

Supplementary Table9 Univariable and multivariable Cox regression model analyses of overall survival in bladder cancers with 3-4sites metastasis after PSM

| Variables | level |  |  |  |  |  |  |
| --- | --- | --- | --- | --- | --- | --- | --- |
|  |  | P | HR | 95%CI | P | HR | 95%CI |
| Age at diagnosis(years) | <70 | Ref |  |  |  |  |  |
|  | >=70 | 0.246 | 1.267 | 0.849-1.891 |  |  |  |
| Race | White | Ref |  |  |  |  |  |
|  | Black | 0.161 | 1.548 | 0.840-2.852 |  |  |  |
|  | Other | 0.790 | 0.893 | 0.387-2.058 |  |  |  |
| Sex | Female | Ref |  |  |  |  |  |
|  | Male | 0.336 | 0.799 | 0.506-1.262 |  |  |  |
| Primary Site | Trigone of bladder | Ref |  |  |  |  |  |
|  | Lateral wall of bladder | 0.461 | 0.659 | 0.218-1.995 | 0.886 | 0.919 | 0.290-2.914 |
|  | Anterior wall of bladder | 0.563 | 0.605 | 0.110-3.323 | 0.222 | 0.340 | 0.060-1.921 |
|  | Posterior wall of bladder | 0.834 | 0.876 | 0.254-3.021 | 0.542 | 0.678 | 0.194-2.365 |
|  | Bladder neck | **0.024** | 0.171 | 0.037-0.796 | 0.442 | 0.529 | 0.104-2.682 |
|  | Ureteric orifice | **0.026** | 0.180 | 0.039-0.818 | 0.216 | 0.371 | 0.077-1.784 |
|  | Overlapping lesion of bladder | 0.464 | 0.655 | 0.210-2.036 | 0.269 | 0.518 | 0.161-1.663 |
|  | Bladder, NOS | 0.306 | 0.586 | 0.211-1.629 | 0.275 | 0.562 | 0.199-1.583 |
| Histologic type | Non-Papillary Transitional cell carcinoma | Ref |  |  |  |  |  |
|  | Papillary Transitional cell carcinoma | 0.451 | 1.220 | 0.727-2.046 |  |  |  |
|  | Squamous cell neoplasms | 0.425 | 1.454 | 0.580-3.649 |  |  |  |
|  | Adenomas and adenocarcinoma | 0.692 | 1.187 | 0.507-2.77 |  |  |  |
|  | Other | 0.901 | 1.041 | 0.554-1.954 |  |  |  |
| Grade | II | Ref |  |  |  |  |  |
|  | III | 0.995 | 1.002 | 0.463-2.169 |  |  |  |
|  | IV | 0.528 | 0.786 | 0.372-1.659 |  |  |  |
| T stage | T0+T1 | Ref |  |  |  |  |  |
|  | T2 | 0.536 | 0.791 | 0.377-1.661 |  |  |  |
|  | T3 | 0.351 | 0.599 | 0.204-1.759 |  |  |  |
|  | T4 | 0.353 | 0.677 | 0.297-1.542 |  |  |  |
|  | TX + unknown | 0.290 | 0.683 | 0.343-1.363 |  |  |  |
| N stage | N0 | Ref |  |  |  |  |  |
|  | N1 | 0.625 | 1.203 | 0.573-2.526 |  |  |  |
|  | N2 | 0.537 | 1.214 | 0.656-2.244 |  |  |  |
|  | N3 | 0.719 | 0.826 | 0.292-2.337 |  |  |  |
|  | NX + unknown | 0.548 | 0.865 | 0.540-1.386 |  |  |  |
| Surgery | No | Ref |  |  |  |  |  |
|  | Yes | **0.020** | 0.596 | 0.385-0.923 | 0.138 | 0.672 | 0.398-1.136 |
| Surgery about regional lymph nodes | Surgery but no LNs removed | Ref |  |  |  |  |  |
|  | No surgery | **0.031** | 1.627 | 1.045-2.534 |  |  |  |
|  | Surgery and LN removed | 0.484 | 0.740 | 0.318-1.721 |  |  |  |
| Radiotherapy | Yes |  |  |  |  |  |  |
|  | None/Unknown | **<0.0001** | 2.372 | 1.482-3.794 | 0.628 | 1.222 | 0.543-2.749 |
| Radiation sequence with surgery | No radiotherapy and/or surgery | Ref |  |  |  |  |  |
|  | Radiation after surgery | <0.0001 | 0.322 | 0.182-0.568 |  |  |  |
|  | other | 0.173 | 0.370 | 0.089-1.544 |  |  |  |
| Chemotherapy | No/Unknown |  |  |  |  |  |  |
|  | Yes | **<0.0001** | 0.287 | 0.178-0.462 | **<0.0001** | 0.267 | 0.155-0.458 |
| Metastases sites including liver | No | Ref |  |  |  |  |  |
|  | Yes | 0.073 | 2.133 | 0.931-4.883 |  |  |  |
| Metastases sites including brain | No | Ref |  |  |  |  |  |
|  | Yes | 0.842 | 0.953 | 0.597-1.524 |  |  |  |

Supplementary Table10 Univariable and multivariable Cox regression model analyses of overall survival in bladder cancers with bone metastasis after PSM

| Variables | level |  |  |  |  |  |  |
| --- | --- | --- | --- | --- | --- | --- | --- |
|  |  | P | HR | 95%CI | P | HR | 95%CI |
| Age at diagnosis(years) | <70 | Ref |  |  |  |  |  |
|  | >=70 | 0.198 | 1.154 | 0.928-1.436 |  |  |  |
| Race | White | Ref |  |  |  |  |  |
|  | Black | 0.142 | 1.271 | 0.923-1.752 |  |  |  |
|  | Other | 0.969 | 1.010 | 0.618-1.650 |  |  |  |
| Sex | Female | Ref |  |  |  |  |  |
|  | Male | **0.045** | 0.780 | 0.611-0.995 | 0.550 | 0.925 | .0.715-1.195 |
| Primary Site | Trigone of bladder | Ref |  |  |  |  |  |
|  | Dome of bladder | 0.686 | 1.172 | 0.543-2.528 |  |  |  |
|  | Lateral wall of bladder | 0.581 | 1.199 | 0.629-2.286 |  |  |  |
|  | Anterior wall of bladder | 0.976 | 0.983 | 0.328-2.947 |  |  |  |
|  | Posterior wall of bladder | 0.747 | 1.121 | 0.560-2.243 |  |  |  |
|  | Bladder neck | 0.517 | 0.788 | 0.384-1.618 |  |  |  |
|  | Ureteric orifice | 0.955 | 1.043 | 0.239-4.555 |  |  |  |
|  | Overlapping lesion of bladder | 0.581 | 1.166 | 0.675-2.015 |  |  |  |
|  | Bladder, NOS | 0.854 | 1.049 | 0.628-1.752 |  |  |  |
| Histologic type | Non-Papillary Transitional cell carcinoma | Ref |  |  |  |  |  |
|  | Papillary Transitional cell carcinoma | 0.260 | 0.859 | 0.658-1.120 | 0.556 | 0.920 | 0.699-1213 |
|  | Squamous cell neoplasms | **<0.0001** | 2.429 | 1.490-3.960 | **0.008** | 1.958 | 1.194-3.211 |
|  | Adenomas and adenocarcinoma | **0.030** | 0.566 | 0.338-0.946 | 0.075 | 0.625 | 0.373-1.048 |
|  | Other | **0.001** | 2.105 | 1.335-3.319 | **<0.0001** | 2.372 | 1.491-3.772 |
| Grade | II | Ref |  |  |  |  |  |
|  | III | 0.847 | 1.041 | 0.692-1.566 |  |  |  |
|  | IV | 0.665 | 0.917 | 0.618-1.359 |  |  |  |
| T stage | T0+T1 | Ref |  |  |  |  |  |
|  | T2 | 0.408 | 1.152 | 0.824-1.612 |  |  |  |
|  | T3 | 0.484 | 0.828 | 0.489-1.403 |  |  |  |
|  | T4 | 0.535 | 1.117 | 0.788-1.584 |  |  |  |
|  | TX + unknown | 0.063 | 1.409 | 0.982-2.020 |  |  |  |
| N stage | N0 | Ref |  |  |  |  |  |
|  | N1 | 0.446 | 1.182 | 0.769-1.815 |  |  |  |
|  | N2 | 0.460 | 1.123 | 0.826-1.525 |  |  |  |
|  | N3 | 0.122 | 1.501 | 0.898-2.511 |  |  |  |
|  | NX + unknown | 0.253 | 1.183 | 0.887-1.580 |  |  |  |
| Surgery | No | Ref |  |  |  |  |  |
|  | Yes | **0.007** | 0.717 | 0.564-0.912 | **0.002** | 0.679 | 0.530-0.870 |
| 0.870Surgery about regional lymph nodes | Surgery but no LNs removed | Ref |  |  |  |  |  |
|  | No surgery | **0.024** | 1.322 | 1.038-1.683 |  |  |  |
|  | Surgery and LN removed | **0.012** | 0.485 | 0.275-0.854 | **0.013** | 0.486 | 0.275-0.858 |
| Radiotherapy | Refused | Ref |  |  |  |  |  |
|  | Yes | 0.402 | 0.681 | 0.277-1.674 |  |  |  |
|  | None/Unknown | 0.429 | 0.699 | 0.288-1.699 |  |  |  |
| Radiation sequence with surgery | No radiotherapy and/or surgery | Ref |  |  |  |  |  |
|  | Radiation after surgery | 0.360 | 0.880 | 0.670-1.156 |  |  |  |
|  | Radiation before surgery | 0.160 | 0.368 | 0.091-1.484 |  |  |  |
| Chemotherapy | No/Unknown | Ref |  |  |  |  |  |
|  | Yes | **<0.0001** | 0.475 | 0.372-0.607 | **<0.0001** | 0.465 | 0.362-0.599 |

Supplementary Table11 Univariable and multivariable Cox regression model analyses of overall survival in bladder cancers with lung metastasis after PSM

| Variables | level |  |  |  |  |  |  |
| --- | --- | --- | --- | --- | --- | --- | --- |
|  |  | P | HR | 95%CI | P | HR | 95%CI |
| Age at diagnosis(years) | <70 | Ref |  |  |  |  |  |
|  | >=70 | **0.003** | 1.448 | 1.139-1.841 | 0.275 | 1.151 | 0.894-1.480 |
| Race | White | Ref |  |  |  |  |  |
|  | Black | 0.832 | 1.034 | 0.758-1.410 |  |  |  |
|  | Other | 0.510 | 0.808 | 0.429-1.522 |  |  |  |
| Sex | Female | Ref |  |  |  |  |  |
|  | Male | **0.040** | 0.776 | 0.609-0.988 | 0.185 | 0.844 | 0.656-1.085 |
| Primary Site | Trigone of bladder | Ref |  |  |  |  |  |
|  | Dome of bladder | 0.071 | 0.345 | 0.109-1..094 |  |  |  |
|  | Lateral wall of bladder | 0.475 | 0.761 | 0.360-1.609 |  |  |  |
|  | Anterior wall of bladder | 0.326 | 0.631 | 0.252-1.583 |  |  |  |
|  | Posterior wall of bladder | 0.507 | 1.302 | 0.597-2.837 |  |  |  |
|  | Bladder neck | 0.562 | 0.775 | 0.328-1.831 |  |  |  |
|  | Ureteric orifice | 0.641 | 0.77 | 0.270-2.240 |  |  |  |
|  | Ureteric orifice | 0.050 | 0.220 | 0.048-0.999 |  |  |  |
|  | Overlapping lesion of bladder | 0.875 | 0.950 | 0.498-1.810 |  |  |  |
|  | Bladder, NOS | 0.880 | 1.048 | 0.569-1.933 |  |  |  |
| Histologic type | Non-Papillary Transitional cell carcinoma | Ref |  |  |  |  |  |
|  | Papillary Transitional cell carcinoma | **<0.001** | 0.583 | 0.441-0.769 | **0.001** | 0.607 | 0.457-0.806 |
|  | Squamous cell neoplasms | 0.121 | 1.390 | 0.916-2.109 | **0.019** | 1.680 | 1.088-2.596 |
|  | Adenomas and adenocarcinoma | 0.060 | 0.455 | 0.200-1.035 | 0.291 | 0.636 | 0.274-1.473 |
|  | Other | 0.902 | 0.976 | 0.665-1.433 | 0.406 | 0.847 | 0.572-1.254 |
| Grade | II | Ref |  |  |  |  |  |
|  | III | 0.472 | 1.157 | 0.777-1.724 |  |  |  |
|  | IV | 0.429 | 1.165 | 0.797-1.704 |  |  |  |
| T stage | T0 | Ref |  |  |  |  |  |
|  | T1 | 0.288 | 0.340 | 0.046-2.486 |  |  |  |
|  | T2 | 0.462 | 0.476 | 0.0663.436 |  |  |  |
|  | T3 | 0.361 | 0.393 | 0.053-2.913 |  |  |  |
|  | T4 | 0.393 | 0.422 | 0.058-3.049 |  |  |  |
|  | TX + unknown | 0.405 | 0.431 | 0.060-3.121 |  |  |  |
| N stage | N0 | Ref |  |  |  |  |  |
|  | N1 | 0.225 | 1.257 | 0.869-1.818 |  |  |  |
|  | N2 | 0.750 | 1.061 | 0.739-1.523 |  |  |  |
|  | N3 | 0.244 | 0.682 | 0.358-1.298 |  |  |  |
|  | NX + unknown | 0.565 | 1.092 | 0.809-1.475 |  |  |  |
| Surgery | No | Ref |  |  |  |  |  |
|  | Yes | **0.003** | 0.656 | 0.499-0.863 | 0.101 | 0.786 | 0.586-1.048 |
| Surgery about regional lymph nodes | Surgery but no LNs removed | Ref |  |  |  |  |  |
|  | No surgery | **0.019** | 1.393 | 1.055-1.839 |  |  |  |
|  | Surgery and LN removed | **0.002** | 0.495 | 0.317-0.774 | **0.018** | 0.573 | 0.361-0.909 |
| Radiotherapy | Refused | Ref |  |  |  |  |  |
|  | Yes | 0.810 | 0.892 | 0.352-2.262 |  |  |  |
|  | None/Unknown | 0.934 | 0.963 | 0.396-2.341 |  |  |  |
| Radiation sequence with surgery | No radiotherapy and/or surgery | Ref |  |  |  |  |  |
|  | Radiation after surgery | 0.375 | 0.836 | 0.562-1.243 |  |  |  |
|  | Radiation before surgery | 0.438 | 0.576 | 0.143-2.320 |  |  |  |
| Chemotherapy | No/Unknown | Ref |  |  |  |  |  |
|  | Yes | **<0.0001** | 0.410 | 0.315-0.535 | **<0.0001** | 0.447 | 0.339-0.590 |

Supplementary Table12 Univariable and multivariable Cox regression model analyses of overall survival in bladder cancers with liver metastasis after PSM

| Variables | level |  |  |  |  |  |  |
| --- | --- | --- | --- | --- | --- | --- | --- |
|  |  | P | HR | 95%CI | P | HR | 95%CI |
| Age at diagnosis(years) | <70 | Ref |  |  |  |  |  |
|  | >=70 | **0.006** | 1.603 | 1.149-2.238 | **0.034** | 1.443 | 1.028-2.025 |
| Race | White | Ref |  |  |  |  |  |
|  | Black | 0.552 | 1.166 | 0.728-1.868 |  |  |  |
|  | Other | 0.707 | 0.864 | 0.403-1.851 |  |  |  |
| Sex | Female | Ref |  |  |  |  |  |
|  | Male | 0.915 | 1.019 | 0.723-1.437 |  |  |  |
| Primary Site | Trigone of bladder | Ref |  |  |  |  |  |
|  | Dome of bladder | 0.381 | 0.566 | 0.158-2.022 |  |  |  |
|  | Lateral wall of bladder | 0.907 | 0.950 | 0.398-2.264 |  |  |  |
|  | Anterior wall of bladder | 0.246 | 0.510 | 0.163-1.590 |  |  |  |
|  | Posterior wall of bladder | 0.345 | 1.478 | 0.657-3.327 |  |  |  |
|  | Bladder neck | 0.481 | 1.428 | 0.530-3.846 |  |  |  |
|  | Ureteric orifice | 0.142 | 0.314 | 0.067-1.476 |  |  |  |
|  | Ureteric orifice | 0.090 | 0.171 | 0.022-1.320 |  |  |  |
|  | Overlapping lesion of bladder | 0.697 | 1.145 | 0.580-2.262 |  |  |  |
|  | Bladder, NOS | 0.546 | 0.829 | 0.452-1.523 |  |  |  |
| Histologic type | Non-Papillary Transitional cell carcinoma | Ref |  |  |  |  |  |
|  | Papillary Transitional cell carcinoma | 0.083 | 0.714 | 0.488-1.045 |  |  |  |
|  | Squamous cell neoplasms | 0.856 | 0.934 | 0.449-1.944 |  |  |  |
|  | Adenomas and adenocarcinoma | 0.444 | 0.772 | 0.398-1.498 |  |  |  |
|  | Other | 0.791 | 1.060 | 0.689-1.631 |  |  |  |
| Grade | II | Ref |  |  |  |  |  |
|  | III | 0.339 | 1.321 | 0.747-2.337 |  |  |  |
|  | IV | 0.987 | 0.995 | 0.560-1.768 |  |  |  |
| T stage | T1 | Ref |  |  |  |  |  |
|  | T2 | 0.298 | 0.775 | 0.480-1.253 |  |  |  |
|  | T3 | 0.751 | 0.889 | 0.430-1.836 |  |  |  |
|  | T4 | 0.812 | 0.940 | 0.565-1.565 |  |  |  |
|  | TX + unknown | 0.321 | 0.787 | 0.491-1.262 |  |  |  |
| N stage | N0 | Ref |  |  |  |  |  |
|  | N1 | 0.422 | 0.743 | 0.359-1.536 |  |  |  |
|  | N2 | 0.074 | 0.661 | 0.420-1.041 |  |  |  |
|  | N3 | 0.590 | 1.237 | 0.571-2.682 |  |  |  |
|  | NX + unknown | 0.127 | 0.742 | 0.506-1.088 |  |  |  |
| Surgery | No | Ref |  |  |  |  |  |
|  | Yes | 0.230 | 0.816 | 0.585-1.137 | **0.005** | 0.600 | 0.421-0.855 |
| Surgery about regional lymph nodes | Surgery but no LNs removed | Ref |  |  |  |  |  |
|  | No surgery | 0.260 | 1.212 | 0.867-1.694 |  |  |  |
|  | Surgery and LN removed | 0.622 | 0.798 | 0.325-1.959 |  |  |  |
| Radiotherapy | Refused | Ref |  |  |  |  |  |
|  | None/Unknown | 0.843 | 1.051 | 0.643-1.718 |  |  |  |
| Radiation sequence with surgery | No radiotherapy and/or surgery | Ref |  |  |  |  |  |
|  | Radiation after surgery | 0.926 | 1.026 | 0.592-1.779 |  |  |  |
| Chemotherapy | No/Unknown | Ref |  |  |  |  |  |
|  | Yes | **<0.0001** | 0.456 | 0.319-0.650 | <0.0001 | 0.415 | 0.283-0.608 |

Supplementary Table13 Univariable and multivariable Cox regression model analyses of overall survival in bladder cancers with LN metastasis after PSM

| Variables | level |  |  |  |  |  |  |
| --- | --- | --- | --- | --- | --- | --- | --- |
|  |  | **P** | HR | 95%CI | P | HR | 95%CI |
| Age at diagnosis(years) | <70 |  |  |  |  |  |  |
|  | >=70 | 0.111 | 1.814 | 0.872-3.775 |  |  |  |
| Race | White |  |  |  |  |  |  |
|  | Black | 0.275 | 0.517 | 0.158-1.690 |  |  |  |
|  | Other | 0.597 | 0.584 | 0.080-4.287 |  |  |  |
| Sex | Female |  |  |  |  |  |  |
|  | Male | 0.174 | 0.630 | 0.323-1.227 |  |  |  |
| Primary Site | Trigone of bladder |  |  |  |  |  |  |
|  | Lateral wall of bladder | 0.880 | 0.830 | 0.074-9.311 |  |  |  |
|  | Posterior wall of bladder | 0.951 | 4.651 | 0.056-14.666 |  |  |  |
|  | Bladder neck | 0.756 | 0.643 | 0.040-10.348 |  |  |  |
|  | Overlapping lesion of bladder | 0.891 | 1.134 | 0.140-9.208 |  |  |  |
|  | Bladder, NOS | 0.891 | 1.153 | 0.151-8.800 |  |  |  |
| Histologic type | Non-Papillary Transitional cell carcinoma |  |  |  |  |  |  |
|  | Papillary Transitional cell carcinoma | **0.038** | 0.280 | 0.084-0.929 | **0.045** | 0.289 | 0.086-0.975 |
|  | Squamous cell neoplasms | 0.599 | 1.384 | 0.413-4.644 | 0.333 | 0.841 | 0.535-6.330 |
|  | Adenomas and adenocarcinoma | 0.243 | 2.387 | 0.555-10.267 | 0.341 | 2.054 | 0.467-9.038 |
| Grade | II |  |  |  |  |  |  |
|  | III | 0.399 | 2.492 | 0.299-20.774 |  |  |  |
|  | IV | 0.374 | 2.473 | 0.336-18.208 |  |  |  |
| Surgery | No |  |  |  |  |  |  |
|  | Yes | 0.391 | 0.732 | 0.359-1.492 | 0.119 | 0.537 | 0.246-1.174 |
| Surgery about regional lymph nodes | Surgery but no LNs removed |  |  |  |  |  |  |
|  | No surgery | 0.460 | 1.316 | 0.636-2.724 |  |  |  |
|  | Surgery and LN removed | 0.659 | 0.761 | 0.226-2.560 |  |  |  |
| Radiotherapy | Yes |  |  |  |  |  |  |
|  | None/Unknown | 0.723 | 1.295 | 0.310-5.415 |  |  |  |
| Radiation sequence with surgery | No radiotherapy and/or surgery |  |  |  |  |  |  |
|  | Radiation after surgery | 0.997 | 0.996 | 0.134-7.386 |  |  |  |
| Chemotherapy | No/Unknown |  |  |  |  |  |  |
|  | Yes | **<0.0001** | 0.309 | 0.134-0.711 | **0.002** | 0.223 | 0.087-0.572 |
